# Anelastic Effects in Fe–Ga and Fe–Ga-Based Alloys: A Review

**DOI:** 10.3390/ma16062365

**Published:** 2023-03-15

**Authors:** Igor S. Golovin

**Affiliations:** 1 National University of Science and Technology MISIS, Leninsky ave. 4, 119049 Moscow, Russia; i.golovin@misis.ru or i.s.golovin@mospolytech.ru; 2 Moscow Polytechnic University, B. Semenovskay 38, 107023 Moscow, Russia

**Keywords:** Fe–Ga based alloys, anelasticity, internal friction, damping capacity, thermally activated, transient and hysteretic effects, first- and second-order phase transitions, in situ neutron diffraction, magnetostriction

## Abstract

Fe–Ga alloys (GalFeNOLs) are the focus of attention due to their enhanced magneto-elastic properties, namely, magnetostriction in low saturation magnetic fields. In the last several years, special attention has been paid to the anelastic properties of these alloys. In this review, we collected and analyzed the frequency-, amplitude-, and temperature-dependent anelasticity in Fe–Ga and Fe–Ga-based alloys in the Hertz range of forced and free-decay vibrations. Special attention is paid to anelasticity caused by phase transitions: for this purpose, in situ neutron diffraction tests with the same heating or cooling rates were carried out in parallel with temperature dependencies measurements to control ctructure and phase transitions. The main part of this review is devoted to anelastic effects in binary Fe–Ga alloys, but we also consider ternary alloys of the systems Fe–Ga–Al and Fe–Ga–RE (RE—Rare Earth elements) to discuss similarities and differences between anelastic properties in Fe–Ga and Fe–Al alloys and effect of RE elements. We report and discuss several thermally activated effects, including Zener- and Snoek-type relaxation, several transient anelastic phenomena caused by phase transitions (D0_3_ ↔ A2, D0_3_ → L1_2_, L1_2_ ↔ D0_19_, D0_19_ ↔ B2, Fe_13_Ga_9_ → L_12_+Fe_6_Ga_5_ phases), and their influence on the above-mentioned thermally activated effects. We also report amplitude-dependent damping caused by dislocations and magnetic domain walls and try to understand the paradox between the Smith–Birchak model predicting higher damping capacity for materials with higher saturation magnetostriction and existing experimental results. The main attention in this review is paid to alloys with 17–20 and 25–30%Ga as the alloys with the best functional (magnetostriction) properties. Nevertheless, we provide information on a broader range of alloys from 6 to 45%Ga. Due to the limited space, we do not discuss other mechanical and physical properties in depth but focus on anelasticity. A short introduction to the theory of anelasticity precedes the main part of this review of anelastic effects in Fe–Ga and related alloys and unsolved issues are collected in summary.

## 1. Introduction

In the introduction, we provide only minimum general information on Fe–Ga alloys, which can be relatively easily found in the literature (e.g., [1,2,3,4,5,6,7,8,9,10,11]). Here, we focus the readers’ attention on their essential features, which are important for the discussion of anelastic effects, and on the background of different anelastic effects observed in Fe–Ga-based alloys.

The addition of non-magnetic Ga to iron greatly enhances its saturation magnetostriction in the [001] direction; Fe–Ga alloys (GalFeNOLs) show mechanical properties superior to other magnetostrictive materials, weak dependence of (3/2) λ_100_ on temperature, and a relatively low saturation magnetic field [9]. The combination of these unique characteristics makes the alloys promising in applications such as sensors, actuators, energy harvesters, and spintronic devices working in the kHz range of frequencies. They exhibit very low hysteresis and high tensile strength (~500 MPa), while their magnetomechanical properties have limited dependence on the range of climatic temperatures.

The magnetostriction (λ) of single Fe–Ga crystals approaches 400 ppm along the <100> direction [1]. The tetragonal magnetostriction of polycrystalline Fe–Ga exhibits 2 peaks near 19% and 27%Ga (in this paper, we use at.%; if not, it is written “wt.%”) [2,3]. According to Smith and Birchak’s theory [5], the maximal value of the damping capacity in ferromagnetic materials is proportional to saturation magnetostriction, λ_S_: Q_max_^−1^ ~ λ_S_. Thus, Fe–Ga alloys are also candidates for damping applications [4], along with the Fe–Al, Fe–Cr, and Fe–Mo alloys used as high-damping materials with the magnetomechanical mechanism of damping [6].

In several publications [7], it is believed that the increase in magnetostriction in Fe–Ga alloys is due to the preferential (110) Ga–Ga pairing in the disordered body-centered cubic (bcc) structure, which is determined either from splitting of XRD peaks or by TEM. Careful analysis of published XRD data suggests that such a peak splitting should take place along with the appearance of superstructural peaks, which never were reported leaving the problem open until now [12,13].

Fe–Ga alloys are also known for their ordering of Ga atoms in fcc (A1), bcc (A2), and hcp (A3) iron: the type of order depends on temperature range and %Ga atoms. Most recently, the equilibrium Fe–Ga phase diagram proposed by T. Gödecke and W. Köster [14] and adopted by O. Kubaschewski [15] was corrected in the temperature range below 600 °C to extend the range of the L1_2_ phase [16,17].

In a wide range of Ga concentrations, the key role in the formation of desirable functional properties is the transition between metastable D0_3_ and equilibrium fcc L1_2_ ordered phases below 610 °C [18].

**Figure 1:**
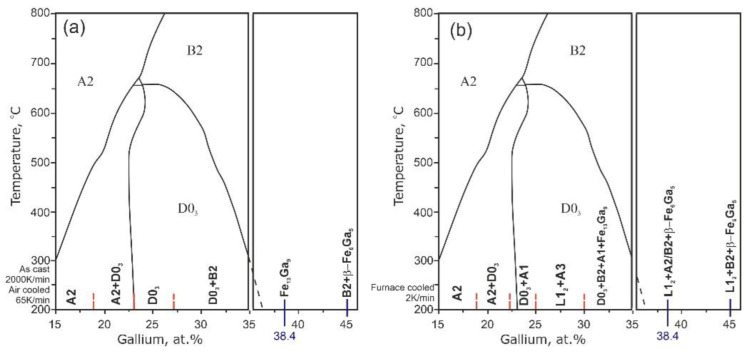
Metastable phase diagram by Ikeda et al. [8] supplied with structures at room temperature by Balagurov et al. [19] for Fe–(15–45)%Ga alloys after casting with a cooling rate ≈ 2000 K/min, cooling in the air at ≈ 60 K/min (**a**), and furnace cooling at 900 °C with 2 K/min (**b**).

The structure of the as-cast, furnace- and air-cooled Fe–Ga alloys is very different compared with that predicted by equilibrium phase diagrams due to sluggish Ga atoms diffusion. In many cases, phase transitions develop in accordance with the *metastable* diagram proposed by Ikeda et al. [8]. Thus, the metastable phase diagram with two body-centered cubic (bcc)-derived ordered phases, B2 and D0_3_, describes the structure of Fe–Ga alloys far better as it was proved in for Fe–(9–33)%Ga [19] (Figure 1). Time-temperature-transition (TTT) diagrams for the decay of high-temperature disordered solid solution (A2) were built for several Fe–Ga alloys using different experimental techniques, including in situ neutron diffraction measurements [20].

**Figure 2:**
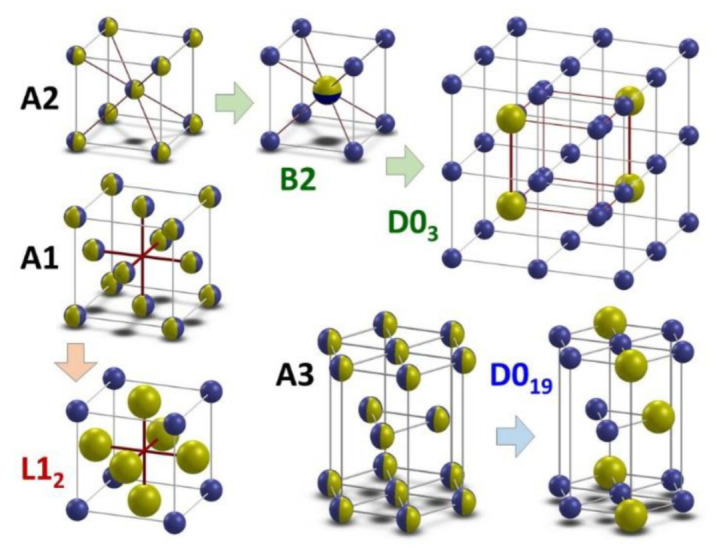
Typical disordered (A1, A2, and A3) and ordered (B2, D0_3_, L1_2_, and D0_19_) phases in Fe–Ga alloys. Colors of balls correspond to composition Fe_3_Ga.

According to the equilibrium phase diagrams and our own studies, the following structures are observed in Fe–Ga alloys at different temperatures in the concentration range below roughly 30 at.% (Figure 2):

- The **A1**—has an γ-Fe-type structure with Fe and Ga (or Al) atoms randomly distributed, sp. gr. *Fm*3*m* (N225);
- The **A2**—has an α-Fe-type structure with Fe and Ga (or Al) atoms randomly distributed, sp. gr. *Im*3*m* (N229);
- The A3—has a Mg-type structure with randomly distributed Ga atoms, sp. gr. *P*6_3_/*mmc* (N194);
- The L1_2_—has an Cu_3_Au-type structure with Fe and Ga atoms partially ordered, sp. gr. *Fm*3*m* (result of A1 phase ordering) (N226);
- The **B2**—has a CsCl-type structure with Fe and Me atoms partially ordered, sp. gr. *Pm*3*m* (result of A2 phase ordering) (N221);
- The **D0_3_**—has a BiF_3_-type structure with Fe and Me atoms partially ordered, sp. gr. *Fm*3*m* (result of A2 phase ordering) (225);
- The D0_19_—has a MgCd_3_-type structure with Fe and Ga/Ge atoms partially ordered, sp. gr. *P*6_3_/*mmc* (result of A3 phase ordering) (N194).

Phases also observed in Fe–Al alloys are underlined in **bold** font. The ground-state electron configurations of nonmagnetic elements Al and Ga are 1s^2^2s^2^2p^6^3s^2^3p^1^ and 1s^2^2s^2^2p^6^3s^2^3p^6^3d^10^4s^2^4p^1^. Their outer-shell electron configurations are similar because the d-shell electrons of Ga are filled, and the d-shell electrons of Al are absent. Both Al and Ga enhance the magnetostriction of bcc Fe, making a magnetoelastic contribution to damping capacity.

**Figure 3:**
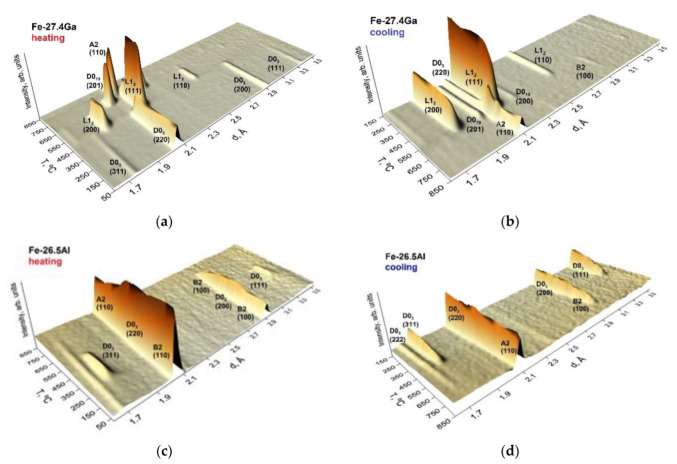
The 3D visualization of in situ neutron diffraction pattern evolution for Fe–27.4Ga (**a**,**b**) and Fe–26.5Al (**c**,**d**) water quenched samples at continuous heating (**a**,**c**) and cooling from 850 °C (**b**,**d**) [24].

In situ 3D neutron diffraction patterns for water-quenched Fe–27.4Ga and Fe–26.5Al alloys at continuous heating with 2 K/min are presented in Figure 3 [10]. Upon continuous heating, several phase transitions occur according to the neutron diffraction and agreement with existing phase diagrams.

In (Fe–25–28)Ga alloys, the recorded sequence of first-order phase transitions upon heating is D0_3_ → L1_2_ → D0_19_ → B2/A2 (depending on the heating rate: A2 for 2 K/min or B2 for 1 K/min). A second-order A2 → D0_3_ transition precedes the sequence of first-order transitions if only a surface of the directly solidified or quenched samples is analyzed by XRD [21] and position annihilation [22] studies. The range of the D0_3_ → L1_2_ transition depends greatly on the heating rate.

In Fe–(26–27)Al alloys, only second-order transitions—“ordering (of B2 and D0_3_ types) ↔ disordering”—take place. At heating, the recorded sequence of phase transitions for the *water-quenched* sample is B2 → D0_3_ → B2 → A2 (Figure 3a), whereas, in the *furnace-cooled* and *as-cast* alloy, it is D0_3_ → B2 → A2 [23].

Upon continuous cooling from 850 °C, the following phase transitions occur according to neutron diffraction:

- In (Fe–25–28)Ga alloys, the recorded sequence of first-order phase transitions upon cooling is A2(B2) → D0_19_ + L1_2_ (the ratio between these phases depends on the cooling rate as discussed in [20]), and, at the same time, a certain amount of remaining A2 phase gets D0_3_ ordering (Figure 3d);
- In Fe–(26–27)Al alloys, the A2 → B2 → D0_3_ transitions take place.

Atomic arrangement from A2 disordered phase to B2 and D0_3_ (second-order transition, for both systems) or to L1_2_ ordered (through two second-order and one first-order transition in Fe–Ga [21]) phases affects both the value and the sign of magnetostriction coefficients in Fe–Ga. In Fe–Ga alloys with Ga > 30%, another 3 phases were observed in addition to the B2, D0_3_, and L1_2_: Fe_13_Ga_9_ [25] and α- and β-Fe_6_Ga_5_ [26,27].

Aluminum, which is much cheaper than Gallium, can be added to Fe–Ga alloys to substitute some amount of Ga. Substitution of Ga with Al can be made in Fe–Ga-based alloys in certain composition ranges without a substantial reduction in magnetostriction [28]. In ternary Fe–Ga–Al alloys, a substitution of Ga atoms by Al atoms stabilizes the D0_3_ phase at low temperatures and prevents the formation of the L1_2_ phase in alloys with Ga > 20: the D0_3_ phase is an equilibrium low-temperature phase in binary Fe–Al alloys. Fe–Ga–Al alloys have other advantages that include better mechanical strength, high ductility, low-temperature sensitivity, and high-operating temperature capability; these alloys offer a lower cost and high-performance alternative to many of the current magnetostrictive materials used in sensors and actuators [28].

Atomic ordering in both systems—equilibrium long-range D0_3_ in Fe–Al and equilibrium L1_2_ in Fe–Ga—decreases damping due to the pinning of magnetic domain walls at antiphase boundaries. A short range D0_3_ ordering may also take place in both systems under certain conditions: e.g., at slow cooling or at annealing of quenched samples (in Fe–Al, it is known as *K1* range of equilibrium phase diagram). Taking into account that the Fe–Ga metastable diagram is similar to the Fe–Al equilibrium phase diagram, it is not surprising that alloys of both systems have several common features of magnetic and anelastic properties.

Concerning the anelastic properties of Fe–Ga (with 6, 12, and 17%Ga) alloys, to our knowledge, the first paper on damping in Fe–Ga was presented at the 14th International Conference on Internal Friction and Ultrasound Attenuation (ICIFMS-14, Kyoto, 2005) by a group of Japanese and US researchers [4]. It brought this hot topic to specialists. Indeed, this gave a strong push to related research activity on these alloys in China [29] and Russia [30]. Our studies of thermally activated (Snoek and Zener relaxations), transient (due to A2 ↔ D0_3_, D0_3_ → L1_2_, L1_2_ → D0_19_, D0_19_ → B2 phase transitions), and amplitude-dependent (damping) effects in Fe–Ga alloys were strengthened by cooperation with colleagues from China, Spain, Switzerland, Taiwan, and the US. Studies of phase transitions were supported by parallel real-time neutron diffraction measurements at JINR, Dubna [24,30,31,32,33,34,35,36,37,38,39,40,41,42,43,44,45,46,47,48,49,50,51]. A review of frequency- and temperature-dependent anelastic effects in Fe–Ga alloys was presented by the author of this paper at the 18th International Conference on Internal Friction and Mechanical Spectroscopy (ICIFMS-18, Brazil, 2018). Perhaps, it gave an additional stimulus to study anelasticity in Galfrnols. Research groups in China have added several important (D0_3_ → B2 → A2 transition, distinguished between Zener relaxation in D0_3_ and L1_2_ phases, etc.) details and proposed new approaches for better understanding amplitude-dependent damping mechanisms in Fe–Ga alloys [52,53,54,55,56,57]. Most recently, these results were presented at the 19th ICIFMS (2022, Rome) [58,59,60,61,62,63,64]. An interesting analysis of the core loss separation and magnetic Barkhausen noise at the high-frequency range is recently reported for Fe–19Ga–RE alloys by Bircakova et al. [65].

Anelastic temperature- and frequency-dependent effects in Fe–Al alloys were reviewed in [66] and several later publications [67,68,69,70,71,72,73,74,75,76,77,78,79,80,81,82,83,84,85,86,87]. Fe–Al alloys have good damping properties due to magneto-mechanical decay of mechanical vibrations in the Hz range of loading. All studied alloys [88,89,90] were forged, hot rolled, and annealed in high-vacuum furnaces. The maximal damping capacity of the Fe–Al alloys was recorded in the concentration range between 5.5 and 6.2 wt.% of Al, although maximum magnetostriction is observed in the range of 9–12 wt.% Al. This is inconsistent with the well-known Smith and Birchak model [5], which suggests proportionality between damping capacity and saturation magnetostriction for soft magnetic materials. High damping in the Fe–Al alloys has practical importance. It was proved [90] that high damping capacity can be achieved not only in the high-purity laboratory Fe–Al alloys but also in alloys produced using modern highly efficient metallurgical equipment with a heal volume as large as 350 tons. This industrial Fe–Al alloy was found to exhibit high damping, good mechanical properties, and low manufacturing cost [90]. According to the Smith and Birchak (SB) theory, Fe–Ga alloys are also candidates for high damping materials as they have higher saturation magnetostriction compared with Fe–Al alloys.

These papers, findings, similarities, and some contradictions for Fe–Ga and Fe–Al alloys give us the idea to summarize existing results on the anelasticity of Fe–Ga-based alloys. In this review paper, we focus on temperature-, amplitude-, and frequency-dependent anelastic effects in Fe–Ga-based alloys. Specifically, we present the related results of structural studies to clarify the mechanisms of anelastic effects in these alloys. In the next section, we provide a short introduction to the damping mechanisms which were observed in Fe–Ga, Fe–Al, and similar alloys.

## 2. Fundamentals of Anelasticity

Mechanical spectroscopy, referred to as the internal friction (IF) method in the earlier literature, offers special opportunities to study elastic and anelastic phenomena in metals and alloys at the atomic level, providing a response, e.g., from interstitial atoms, vacancies, substitutional atoms, dislocations, grain and magnetic domains boundaries, phase transformations, etc.

***Internal friction*** (after Charles Coulomb [91]) is the capacity of dense materials to transform the energy of mechanical vibrations into heat by different physical processes in the elastic (Hookeian [92]) range of loading. Internal friction is the dissipation of mechanical energy inside a material caused by **an**elasticity—a term introduced by Clarence Zener [93] to distinguish this phenomenon from a broader family of **in**elastic effects in physics. The anelasticity occurs due to a wide range of physical processes in the material and is widely used in solid-state physics, physical metallurgy, and materials science to study structural defects and their mobility, transport phenomena, and phase transformations in solids. In many cases, the highly sensitive and selective spectra of IF contain unique information that cannot be obtained by other methods. In most cases, anelasticity assumes amplitude-independent phenomena, while amplitude-dependent behavior is called damping. Nevertheless, sometimes both terms are applied for both categories—amplitude-dependent and independent effects.

Fundamentals of anelastic behavior of solids, mainly metals, are given in several books: Zener 1948 [93], Mason 1958 [94], Krishtal et al., 1964 [95], Nowick and Berry 1972 [96], De Batist 1972 [97], Postnikov 1974 [98], Lakes 1999 [99], Schaller et al., 2001 [100], Blanter et al., 2007 [101], Ngai [102], and Fang and Jin 2014 [103]. For this reason, these principles are considered here only shortly with respect to the effects found in Fe alloys only.

The variation of anelastic properties of a material depending on time *t* as a result of a relaxation process is known as the Kohlrausch function [104], which is also frequently called the stretched exponential function:

ϕ*_K_*(*t*) *=* exp[(−*t*/τ)^1−*n*^], (1)

where τ is the relaxation time and *n* is the coupling parameter of correlation between the elementary acts of the relaxation process (0 ≤ *n* < 1).

At *n* = 0, all elementary acts of a relaxation process are independent of one another, and the relaxation process is described by a three-element (two springs and dashpot) rheological Zener model [93]. An example of independent relaxation processes is diffusion jumps of interstitial atoms in the crystal lattice of interstitial solid solutions under the effect of applied cyclic stress (e.g., the Snoek effect [105]). In the case of *n* = 0, the thermally activated relaxation peak of internal friction that corresponds to such a process with a single relaxation time can be evaluated for a standard anelastic solid as a function of the loading frequency.

A mechanical loss peak or a reciprocal quality factor, Q^−1^(ω), in the case of a thermally activated relaxation effect with a single relaxation time—no matter which relaxation mechanism is involved—is well known as described by a Debye equation with respect to frequency (ω):

(2a) $$Q^{-1}\left(\omega \right)=\Delta \frac{\omega \tau }{1+{\left(\omega \tau \right)}^{2}}$$

and frequency-dependent elastic modulus, E(ω):

(2b) $$E\left(\omega \right)=E_{R}\left(1+\Delta \frac{\omega^{2}\tau^{2}}{1+\omega^{2}\tau^{2}}\right)=E_{U}\left(1-\frac{\Delta }{1+\omega^{2}\tau^{2}}\right),$$

where τ is the relaxation time, ∆D(=2Q^−1^) is the relaxation strength, ω=2πf with *f* being the frequency of the mechanical vibrations, and *E_R_* and *E_U_* are the relaxed and unrelaxed modulus, respectively. A peak maximum takes place at ω × τ = 1 and Q_m_^−1^ = ∆/2.

Several anelastic effects in solids are described by the Debye function of frequency: ωτ/(1 + (ωτ)^2^). The relaxation strength (∆) is individual for each physical mechanism. In each case, two values—*τ* and *f*—can be varied in experiments. Consequently, two types of amplitude independent tests can be carried out.

**Figure 4:**
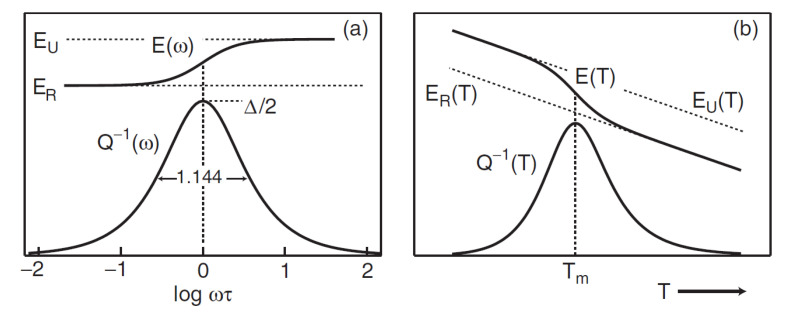
Dynamic modulus E and internal friction *Q*^−1^ of the standard anelastic solid [101]: (**a**) as a function of frequency on a log ωτ scale and (**b**) as a function of temperature at constant frequency. In the latter case, the relaxation-induced step in E(T) is superimposed on the intrinsic temperature dependence of EU(T) and ER(T).

(i) In frequency-dependent IF tests (FDIF), at a fixed temperature (*τ* is a constant in Equation (2) for a given fixed temperature), the frequency *f* is varied over a few orders of magnitude. This method allows direct measurements of *Q*^−1^ and *E* spectra vs. *ω·τ*, as introduced by Equation (2) and shown in Figure 4a [101]. The results can also be presented as a function of *f* (*f* = *ω*/2*π*, as *τ* is a constant for a chosen temperature of measurements).
(ii) Most of the existing mechanical spectroscopy set-ups (e.g., vibrating reeds, torsion pendula) allow measurements of *Q^−^*^1^ as a function of temperature (*T*) but not frequency, i.e., to measure temperature-dependent internal friction (TDIF) (Figure 4b). The temperature dependence (e.g., for jumps of atoms) is typically described by the Arrhenius equation:

(3) $$\tau =\tau_{0}exp\left(\frac{H}{k_{B}T}\right),$$

where *H* is the activation energy (or enthalpy) of the physical phenomenon which controls the relaxation process.

For a fixed frequency and a single relaxation time, the temperature dependence of *Q^−^*^1^ is described by the equation:

(4) $$Q^{-1}\left(T\right)=Q_{m}^{-1}{cosh}^{-1}\left\{\frac{\bar{H}}{k_{B}}\left(\frac{1}{T}-\frac{1}{T_{m}}\right)\right\},$$

where T_m_ and Q_m_^−1^ are the peak temperature and height, respectively, and $\bar{H}$ is the mean value of activation energy. A peak temperature increases with an increase in frequency of measurements in agreement with Equation (3): ω × τ = ω × τ_0_exp(H/k_B_T) = 1.

There are always some defects and local inhomogeneity in real metals and alloys, and thus the relaxation processes with a single relaxation time are rare. In most cases, the distribution of relaxation time is caused either by the distribution in activation energy, H, characteristic relaxation time, τ_0_, or both of them. A variety of particular distribution functions was developed for the description of different alloys. The most widely used one is the Gaussian distribution of a variable z = ln(τ/τ_m_), where $\tau_{m}$ is the mean relaxation time:

(5) $$\Phi \left(z\right)=\frac{exp\left\{-{\left(\frac{z}{\beta_{\tau }}\right)}^{2}\right\}}{\beta_{\tau }\sqrt{\pi }},$$

with β*b_τ_* being a parameter characterizing the width of the relaxation time distribution, and

(6) $$Q^{-1}=\Delta \int_{-\infty }^{\infty }\Phi \left(ln\tau \right)\frac{\omega \tau }{1+{\left(\omega \tau \right)}^{2}}d\left(ln\tau \right)$$

The resulting temperature-dependent damping spectrum is also broader than a single Debye peak:

(7) $$Q^{-1}=Q_{m}^{-1}{cosh}^{-1}\left\{\frac{\bar{H}}{k_{B}r_{2}\left(\beta_{\tau }\right)}\left(\frac{1}{T}-\frac{1}{T_{m}}\right)\right\}$$

Here, r_2_(*β_τ_*) represents the relative peak width, i.e., the peak width with respect to the single Debye peak with *β_τ_* = 0.

The relaxation time distribution (β*b_τ_*) may originate from distributions in both *H* and *τ_0_*. In most cases, these distributions are interrelated:

β*b_τ_* = |β*b*_τ0_
*± βb_H_/*k_B_T|, (8)

values βb_τ0_ and βb_τH_ can be obtained by plotting experimental data in axes βb_τ_ vs. 1/T. If βb_τ_ is temperature independent, peak broadening is due to the distribution in τ_0_; if βb_τ_ is temperature dependent, there is a distribution in activation energy H.

***Thermally activated relaxation phenomena*** are typically described in terms of either frequency or temperature. The above equations are used to evaluate the parameters of an experimental internal friction peak ($\bar{H}$, T_m_, Q_m_^−1^, *β_τ_*), i.e., the parameters of a relaxation mechanism, by fitting the experimental curves. The mean values for the activation energy $\bar{H}$ and the pre-exponential factor *τ*_0_ can be calculated relatively easily from the frequency and/or temperature shift of the peak using the Arrhenius equation. Analytical solutions for more complex cases can be found in the literature (e.g., [96]). A collection of experimental data on anelasticity for different metallic materials can be found in [101,106,107].

The main thermally activated relaxation effects in annealed iron and iron-based alloys are the Snoek relaxation, the Finkelshtein–Rosin relaxation (only in alloys), the Zener relaxation (only in alloys), and grain boundary relaxation [108]. The first three types of relaxation are caused by point defects: either interstitial or substitutional atoms. Point defect relaxation means an anelastic relaxation caused by diffusive jumps of point defects in the field of applied stress in the elastic range of loading. Transient effects in iron-based alloys take place due to first- and second-order phase transitions [10]. In cold-worked alloys, several additional dislocations related anelastic effects may take place, e.g., Bordoni, Hasiguti, and Snoek–Kê–Köster relaxations. The main condition for anelastic relaxation is that the symmetry of the local elastic distortions caused by the defects in the crystal lattice is lower than the symmetry of the lattice itself (so-called selection rule). This causes a reorientation of defects in the applied cyclic stress.

**Figure 5:**
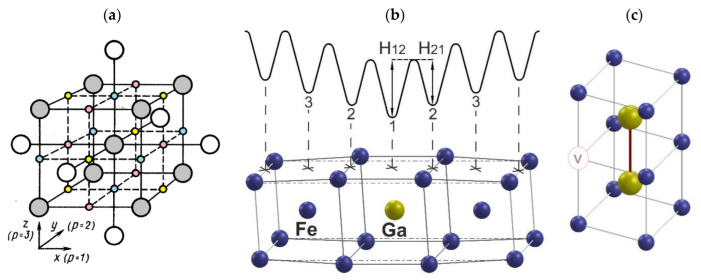
Octahedral interstices in the bcc crystal lattice: large circles are metal atoms; small circles are interstices of the sublattices *p* = 1, 2, and 3 (**a**); scheme of the dependence of the energy of an interstitial atom in octahedral interstices in the (001) plane of *A*2 or *D*0_3_ lattice on the distance from the substitute atom (e.g., Ga) (**b**); and a complex of pair of SA and vacancy (**c**).

The classical ***Snoek relaxation***, originally proposed by Snoek [105] to explain the damping due to C in α-Fe, is an anelastic relaxation caused by ‘heavy’ foreign interstitial atoms (IA) in the bcc metals with body-centered cubic (bcc) lattice. In the case of α-Fe, it is observed in C and N interstitial solid solutions. The interstitial atoms are located in the octahedral interstices of the bcc metal (Figure 5a). The lattice distortions around interstitial atoms, i.e., elastic dipoles, are oriented along one axis (*x*, *y*, or *z*) and have a tetragonal symmetry described by the ***λ***-tensor with two components *λ*_1_ and *λ*_2_ = *λ*_3_, which are not equal to each other. The difference |*λ*_1_ − *λ*_2_| determines the “elastic dipole strength”. By applying alternating stress, the arrangement of interstitial atoms in some octahedral interstices becomes energetically more favorable, and atoms diffuse from one octahedral interstice to the neighboring one just by one stress-induced jump for each interstitial atom. This “diffusion under stress” of interstitial atoms takes place and leads to energy dissipation of mechanical vibrations.

For polycrystalline samples, averaging over all grain orientations gives for flexural vibrations:

Q_m_^−1^ = (0.4 E/9)·[C_0_V·(λ_2_ − λ_1_)^2^ / (k_B_T_m_)], (9)

where *E* is Young’s modulus, *C*_0_ is the atomic fraction of interstitial atoms in the solution, and *V* is the volume of one mole of the host metal.

Substitutional atoms (SA) dissolved in a host lattice of α-Fe influence the original Snoek relaxation via SA-IA interaction in several coordination shells by a change in the activation energy of the atomic jump (Figure 5b) and by a change in the relaxation strength (*λ*_1_ − *λ*_2_) [109]. Some substitutional atoms may not pronouncedly affect the Snoek peak, while others may reduce the peak height and increase the peak width and activation energy leading to the appearance of additional peaks at higher temperature or completely suppress the Snoek peak.

Single substitutional atoms in cubic lattices do not produce an elastic dipole with symmetry lower than that for the host lattice; consequently, they do not produce any relaxation effect under loading. However, the existence of solute next neighbor SA pairs results in an elastic dipole (Figure 5c) and a relaxation maximum under cycling stress, called ***Zener relaxation*** [93]. As only pairs of SA atoms and vacancy are involved in the Zener relaxation mechanism for dilute and random solid solutions, the peak height, which is proportional to the number of reorienting pairs, is ∆_Z_~C_SA_^2^.

Ordering of substitutional solid solution decreases the Zener peak height by a decrease in the number of participation atomic pairs, the reorientation of which would mean disordering of the alloy:

(10) $${Q_{m}}^{-1}\;\propto \;[V0_{\;}f(\chi_{0},C)\;C^{2}(1-C{)}^{2}/(\mathrm{kT})]\sum_{p}(\lambda^{\left(p\right)}{)}^{2},$$

where *V*_0_ is the atomic volume; the coefficients *λ*^(p)^ = (∂*ε*/∂χ_p_)_σ,T_ describe the crystal lattice distortion in the direction *p*, *ε* is the deformation, *χ*_p_ is the number of bonds of one type in the direction *p* (*χ*_p_ is the short-range order parameter), and *χ*_0_ is the value of *χ*_p_ for zero stress *σ*. The function *f*(*χ*_0_,*C*) varies between 0 for the totally ordered and 1 for the totally disordered alloy.

The activation energies of the Zener relaxation in A2, B2, and D0_3_ phases are in the same sequence as those of diffusion, i.e., *H*_A2_ < *H*_B2_ < *H*_D03_ [75].

A thermally activated relaxation peak attributed to grain boundary (GB) sliding [110] is observed in many polycrystalline iron-based alloys at about ½ of their melting temperatures. A variety of possible GB microstructures, a dependence of GB energies on their orientation, and a contribution of segregations of IA or SA at grain boundaries result in distributions of relaxation time in both *H* and *τ*_0_. If Zener and grain boundary relaxations are recorded in an alloy, the Zener peak is located at a low-temperature branch of the GB relaxation which has a much bigger relaxation strength. Snoek, Zener, and GB peaks were recorded in Fe–Al, Fe–Ga, and other α-Fe-based alloys.

***Transient relaxation effects*** (P_Tr_) are another group of anelastic effects which take place due to different phase or structural (e.g., recrystallization) transitions. These effects do not obey the rule ω × τ = 1, which is a signature of thermally activated peaks. The theory of anelasticity for the first- and second-order phase transitions invented by Landau is given in several books on anelasticity, e.g., in the books by Nowick and Berry [96], Benoit [111], and Fantozzi [112]. The maximum of internal friction for both first- and second-order transitions according to Landau’s theory can be described by similar equations:

(11) $$\left\{\begin{array}{c} {fortheIstorder:Q}_{m}^{-1}\left(T_{0}\right)=\frac{\chi^{2}M}{J_{u}\varpi }\\ {forthe2ndorder:Q}_{m}^{-1}\left(T_{C}\right)=\frac{\chi^{2}L}{J_{u}\varpi } \end{array}\right.,$$

where M and L are positive coefficients, ω(=2πf) is frequency, χ is a coupling coefficient between the internal variable (e.g., ordering parameter) and the mechanical stress, and J_U_ is the unrelaxed elastic compliance.

The temperature of transient effects depends on the rate of transition reaching maximum at the temperature at which the transformation rate is maximum, their shape is not described by the Debye equation, and it often is lambda-shaped, λ. Anelasticity due to transient effects $Q_{tr}^{-1}$ for diffusionless first-order transition in many metallic materials are well studied:

(12) $$Q_{tr}^{-1}=\frac{1}{\pi J\sigma_{0}^{2}}\oint \sigma d\varepsilon_{an},$$

where *σ*_0_ is the oscillation amplitude (*σ = σ*_0_cos(*ωt*)) and ε_an_ is the anelastic part of the deformation during one cycle of vibrations.

The transient peak (denoted in this paper as P_Tr_) due to a phase transition, mostly known in the literature to accompany the martensitic transformations in shape memory alloys, is attributed to the transformation rate ∂*n*/∂*t*, where *n* is the amount of the transformed material [113]. The related anelastic strain *dε*_an_ = *k* (∂n/∂*t*) *dt*, which determines the relaxation strength, comes from the lattice deformation when the material is transformed. Delorme and Gobin [114] suggested a “transformation plasticity” approach with the transformation deformation *dε*_p_ = *kσdn* to be linearly dependent on the transformed volume fraction and the applied stress *σ*, leading to

(13) $$Q_{Tr}^{-1}=\frac{k}{J}\frac{dn}{dT}\frac{\dot{T}}{\omega },$$

where k is an empirical coefficient.

Transient peaks are also known as the so-called “$\dot{T}$ effects” due to the dependence of their height on heating or cooling rate [115]. More recent developments of this model have been discussed by San Juan and Pérez-Sáez; practically all proposed models accept the $Q_{Tr}^{-1}\approx \dot{T}/\omega^{n}$ dependence [115]: $Q_{Tr}^{-1}$ increases with an increase in heating or cooling rate, decreases with an increase in frequency, and the peak temperature is independent of frequency. These effects were also recorded in Fe–Al alloys, but they are much better produced in Fe–Ga alloys where not only second but also several first-order transitions were observed [10,11,39]. In contrast with TDIF experiments, the FDIF tests allow excluding transient effects from measured spectra due to $\dot{T}$ = 0.

***Amplitude dependent internal friction*** (ADIF) experiments allow studying the damping capacity of metals and alloys as a function of amplitude of vibrations. High damping metals (“Hidamets” or HDM) are used in various practical applications, such as noise and vibration reduction, preventing fatigue problems, increasing the quality of different cutting tools, etc. In addition to the obvious demand to have a certain level of strength, the Hidamets must have a stable and powerful source of damping and exhibit high damping over a wide frequency and temperature range [6,100,116].

Magneto-mechanical damping (Q_h_^−1^) is one of the four main sources for high damping in metallic materials (thermoelastic martensite, easy movable magnetic domains, high heterogeneity, and easy movable dislocations). Total amplitude-dependent damping in ferromagnetic alloys is composed of non-magnetic (e.g., dislocation) and magneto-mechanical damping: Q^−1^ = Q_d_^−1^ + Q_h_^−1^. Magneto-mechanical damping in ferromagnetic materials has its source in a stress-driven irreversible movement of the magnetic Domain Walls (DWs). Magneto-mechanical damping in Fe alloys at ambient temperatures is typically much higher compared with non-magnetic ones, and it is used as the main source to produce Hidamets. High damping capacity in a wide range of Hz and kHz frequencies is determined by the nonreversible movement of 90° DWs. The dissipated energy depends on DWs’ mobility and density. The mobility of DW depends on (i) the structure and size of magnetic domains and domain boundaries, (ii) the structure of the crystalline lattice in which the DWs move, and (iii) the interaction between the DWs and different imperfections of the crystalline lattice.

A classical review of elastic energy dissipation in ferromagnets was done in [117]. Phenomenologically, the energy loss ∆W due to magnetic domains motion for a vibration stress (σ) below critical stress is well described by the Rayleigh law, ∆W~σ^3^, and damping is proportional to the stress applied: $Q_{h}^{-1}=\Delta W/2\pi W\propto \lambda^{3}E\sigma$, where λ is magnetostriction [5]. With an increase in stress, the dissipated energy saturates (∆W_S_), and damping decreases with increase in applied stress: $Q_{h}^{-1}=\Delta W_{S}/2\pi W=E\Delta W_{S}/\pi \sigma^{2}$ Thus, the amplitude dependence of internal friction $Q_{h}^{-1}\left(\sigma \vee \varepsilon \right)$ has a maximum which was experimentally observed practically in all annealed ferromagnetic alloys. The maximal damping $Q_{h.max}^{-1}$ due to the magneto-mechanical hysteresis is proportional to λ_S_E/σ_i_, where λ_S_ is the saturation magnetostriction and σ_i_ is the average internal stress opposing domain boundary motion. For a Maxwell distribution of internal stresses, the value of maximal hysteretic internal friction (Q_h.max_^−1^) was described by Smith and Birchak [5]:

Q_h.m_^−1^ = 0.34 k λ_S_ E/πσ_i_ ≈ 0.25 k λ_S_ E/πσ_m_, (14)

where σ_m_ is a stress at which ADIF curve has a maximum (σ*_m_* ≈ 0,7σ*_i_*), k ≈ 1 is a constant characteristic of the shape of the hysteresis loop, and E is Young’s modulus.

Thus, according to the Smith and Birchak model, the values of damping should correlate with the values of saturation magnetostriction strain λ_S_, and Fe–Ga alloys with ‘Giant’ magnetostriction (up to 400 ppm in single crystals) are good candidates to Hidamets.

## 3. Anelastic Effects in Binary Fe–Ga and Ternary Fe–Ga-Based Alloys

It would be logical to consider anelastic effects separately for Fe–Ga alloys with Ga concentration below 20–24%Ga and above based on the following reasons: in the former group of alloys, only second-order transitions take place, and in the latter group, both first and second-order transitions. Boundary between these groups cannot be defined precisely as the first-order transitions are controlled by both temperature and time. It was recently proved that long-term aging (300–1800 h) below 600 °C leads to the A2 (or D0_3_) to A1 (or L1_2_) transition even in the alloys with 16–17%Ga [16,17], while in experiments with the constant heating and cooling rate (1–2 K/min), these transitions take place only in the alloys with 24–25%Ga and more [19,49,60,61].

Several dozens of research papers are focused on particular elastic and anelastic effects in different Fe–Ga alloys. The main papers which report anelastic effects in Fe–Ga alloys in a wide range of Ga concentrations used for this review are [39,49,59,60,61]. As a rule of thumb, most authors denoted in their papers the thermally activated effects/peaks as P1, P2, P3, etc., and the transient effects as P_Tr1_, P_Tr2_, P_Tr3_, etc. Obviously, the numbering of these effects in different papers written by different authors is not the same. We did our best to bring clarity into this review to uniform peak numbering, but we cannot completely avoid this problem (some inconsistencies in peak notation); we refer to original figures where these designations are different.

### 3.1. Anelasticity of Fe–Ga Alloys with Ga < 24 at.%

Firstly, TDIF results on Fe–(6,12,17)%Ga were measured (inverted torsion pendulum, free decay, ε_0_ =1 × 10^−5^) and published by Ishimoto et al. in 2006 [4], concluding that “high mechanical loss of the order of 10^−2^ is observed from −190 to 300 °C, which is almost independent of temperature and frequency, and is attributed to magnetoelastic hysteresis”. By applying a static magnetic field of 200 Oe parallel to the torsion axis, Q^−1^(T) was significantly reduced in the entire range of temperature underlying the magneto-mechanical source of damping. The broad internal friction maximum observed at about 100 °C in the Fe–17Ga specimen was tentatively attributed to the Snoek relaxation of carbon in solution. Two Fe–17%Ga alloys were measured and reported at several frequencies between 0.5 and 11 Hz using an inverted torsion pendulum by Fang et al. in 2011 [29] to conclude that “Fe_83_Ga_17_ alloy behaves complicated in the whole temperature range at each frequency. The highest damping value obtained is about 21 × 10^−3^ at 500 °C with f = 8 Hz. For (Fe_83_Ga_17_)_97.25_Cr_2_B_0.75_ sheets, the damping level keeps unchanged until it reaches 400 °C”.

**Figure 6:**
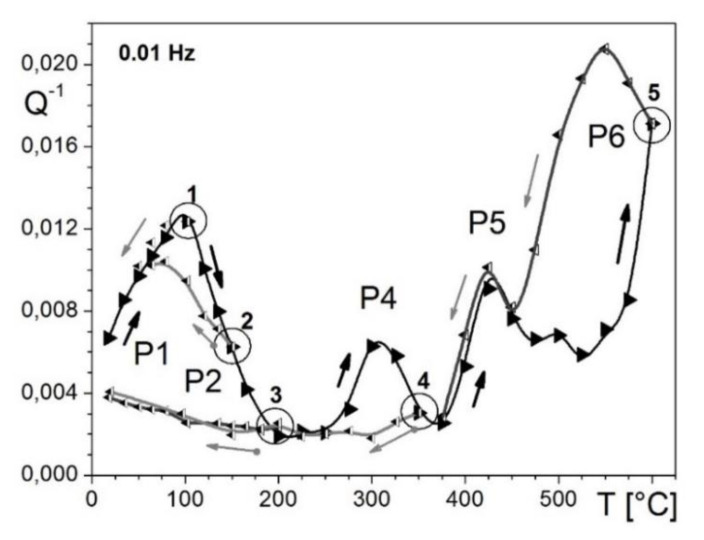
TDIF spectrum of Fe–13Al alloy as plotted from isothermal FDIF curves for f = 0.01 Hz. Arrows indicate whether FDIF tests were carried out with step-by-step increase or decrease in the test temperature [30].

Temperature- and frequency-dependent internal friction spectra for Fe–13Ga were defined in detail in [30] using four different setups. FDIF was measured at a forced torsion pendulum in the frequency range between 50 Hz and 10^−4^ Hz, and temperature from 20 to 600 °C with maximal vibration amplitude was γ_0_ = 5 × 10^−6^. TDIF tests were carried out using (i) DMA Q800 TA (forced vibrations, ε_0_ = 5 × 10^−5^ between −130 and 570 °C), (ii) inverted free-decay torsion pendulum (γ_0_ = 5 × 10^−5^, from −150 to 600 °C with and without applying magnetic field), and (iii) vibrating reed (at 150–3000 Hz). Several IF peaks were recorded, and most of them explained for Fe–13Ga [30] and several Fe–Ga (up to 20%Ga), Fe–Ga–Al and Fe–Ga–Ge alloys [118] (Figure 6):

- A total of 2 broad low-temperature peaks (−80–20 °C, f ≈ 230 Hz and 2 kHz) with activation energy (H) of about 1 eV were tentatively explained as Hasiguti-type relaxations of dislocations with neighboring self-lattice defects. At the same time, authors would not exclude that these effects may correspond to the β and γ relaxations in bcc structures, i.e., kink pair formation in a_0_{111}/2 screw dislocations on {110} and {112} slip planes (these peaks were recorded using TDIF tests, and, therefore, they are not shown in Figure 6). Similar peaks were also recorded in Fe–Al alloys [70];
- Double-headed peaks (P1 and P2) in the temperature range between 50 and 200 °C (dependent on measuring frequency). The peak’s activation energy was estimated from Arrhenius plot as H_P1_ = 0.92–1.08 eV and H_P2_ = 1.0–1.14 eV and characteristic relaxation time (τ_0_) between 10^−15^ and 10^−16^ s (different setups, TDIF tests). These values obtained from FDIF are similar but not the same: H_1_ = 1.1 eV, τ_01_ = 10^−16^ s (P1 effect) and H_2_ = 1.04 eV, τ_02_ = 6 × 10^−14^ s (P2 effect). A similar peak with the activation parameters H_1_ ≈ 0.87 eV and τ_0_ ≈ 10^−16^ s is recorded in Fe–3Ga alloy. These peaks were attributed to the Snoek type relaxation, i.e., stress-induced carbon atom (C) jumps in Fe-C-Fe and Fe_(6-n)_-C-Ga_(n)_ positions (here, *n* is number of Ga atoms in the first coordinate shell of C atom). Similar peaks were also recorded and well resolved in Fe–Al and Fe–Al-Si alloys [80,81];
- The P3 effect above 200 °C was observed only in TDIF tests but never in FDIF tests. This fact underlines that this P3 peak is the transient effect which takes place only at heating due to a structural transition. Similar peaks are denoted below as P_Tr_. It was proposed that the nature of this P3 (or P_Tr1_) peak is due to annihilation of thermal vacancies and ordering (the P3 is not shown in Figure 6, where only results at fixed temperatures are used). Later effects of annihilation of thermal vacancies and ordering were confirmed by positron annihilation and neutron diffraction methods [22,36,59];
- The P4 peak recorded by FDIF tests, similar to the P3 peak at TDIF curves, is unstable with respect to heating and disappears after annealing at 350 °C. The nature of this peak was not explained. We cannot exclude that the P3 and P4 peaks have similar natures, both of them disappearing after heating. Nevertheless, the P4 peak, in contrast with the P3 peak, is a thermally activated effect: its position at FDIF curves depends on measuring temperature in agreement with the ω × τ = 1 rule (see Equation (2a));
- The thermally activated P5 peak at about 450 °C (Figure 6) is stable with respect to heating. Activation parameters of the peak are: τ_05_ ≈ 10^−17^ s and H_5_ = 2.5 eV. According to its activation energy and influence of quenching this peak corresponds to Zener relaxation;
- The P6 peak appears after annealing at about 500 °C and increases strongly with the annealing temperature. Activation parameters (τ_06_ ≈ 2 × 10^−16^ s and H_6_ = 2.7 eV) suggest a grain boundary mechanism of this peak. Later, a similar peak was reported for Fe–27Ga (2.8 eV) [46,47], and, most recently, the interpretation of this effect as a grain boundary peak was confirmed for Fe–30Ga alloy with activation parameters: τ_0_ ≈ 10^−12^ s and H ≈ 2.1 eV [61].

The paper [30] thoroughly covers practically all thermally activated effects in Fe–Ga alloys, which were confirmed in many later papers, apart from several more transient IF effects in alloys with higher concentrations of Ga and, consequently, with first-order transitions.

**Figure 7:**
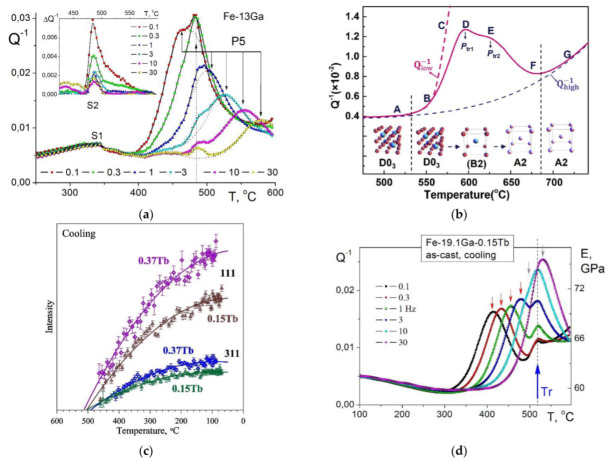
(**a**) Transient (S2) and Zener (P5) IF peaks in Fe–13Ga [30]; (**b**) transient IF peaks (D and E) in Fe–18.3Ga [119]; (**c**) in situ neutron diffraction data suggesting order–disorder transition at ~500 °C (at cooling with 2 K/min); and (**d**) TDIF (cooling with 2 K/min) curves with corresponding PTr peak at ~510 °C [48].

As it concerns temperature-dependent IF, for alloys with less than 20 at.%Ga, it is necessary to mention one more transient effect related to the second-order (ordering and disordering) transition. This transient effect was for the first time reported for the same alloy with 13%Ga used in [30] at 480–490 °C (Figure 7a) [33]. The height of this peak is inversely proportional to the measuring frequency between 0.1 and 30 Hz (Q_m_^−1^ ~ 1/f), which is a typical feature for transient effects. The same transient effect was also recorded for Fe–19%Ga and explained by the D0_3_ ↔ A2 transition [35].

Sun et al. studied this effect in detail using Fe–18.3%Ga [119] and Fe–18Ga–(0.1–1.0)La [52] alloys to report two transient peaks which correspond to the two-stage transition: D0_3_ ↔ B2 ↔ A2 (Figure 6b). Nevertheless, the double shape of the transient peak has not received strong confirmation by structural studies as the appearance of the B2 phase was not recorded during in situ measurements. The presence of the B2 phase was detected only at RT after quenching from different temperatures [119]. It is not excluded that the B2 structure appears during cooling from the temperature range slightly above solvus line as the result of not completing A2 to D0_3_ transition due to inertia at the beginning of cooling process.

The real-time study of this transient effect became possible by parallel in situ measurements of internal friction and neutron diffraction (ND) on the same samples Fe–19.1Ga–Tb with the same heating and cooling rate (Figure 7c,d only shows ND and IF curves at cooling). Thus, it was confirmed that the transient peak at about 500 °C accompanies the D0_3_ ↔ A2 transition recorded by real time ND in the same regime (Figure 7c,d) [48]. Red arrows in Figure 6d indicate positions of thermally activated Zener peak measured at different frequencies. The Zener and transient peaks overlap if the measuring frequency is 10 or 30 Hz. At lower frequencies, they are located in different temperature ranges.

We must note that the appearance and parameters of the anelastic transient effect due to the D0_3_ ↔ A2 transition depend on many factors. It overlaps with Zener thermally activated relaxation; thus, sometimes the transient effect claims itself as a clear peak and TDIF curves, but sometimes it is not clearly manifested and only contributes to total damping, producing an unusual dependence of relaxation strength of the total peak on measuring frequency, i.e., on temperature. These results, either by combination of two different effects (Zener and transient peaks) in the same temperature range or the ordering degree D0_3_ → A2, may influence an increase in Zener relaxation strength in agreement with Equation (10). The effect of disordering on an increase in Zener peak height in Fe–Al alloys was reported in detail in [30].

**Figure 8:**
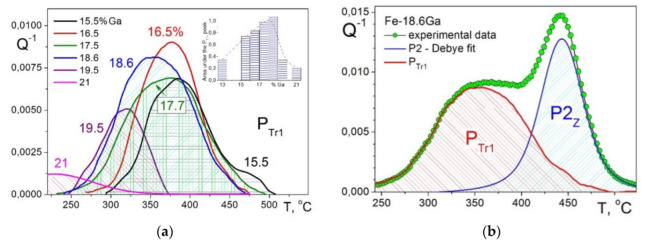
Transient peak P_Tr1_ in water quenched Fe–(16–21)%Ga alloys: inset—area under the peak (**a**), method of deconvolution via fitting P2_Z_ peak (**b**).

All together, we have studied at least a dozen of Fe–Ga compositions with 13 < Ga < 22 with concentration steps less than 2%Ga (sometimes 0.5%) in different structural states: as-cast, as-quenched, annealed, and cold-worked states. Although it became clear that this transient effect is very often observed in water- and air-cooled states, that it is smaller or absent after annealing and was not observed after cold work (only the Fe-16.5%Ga alloy was studied in CW state), it is still difficult to give a systematic picture of this effect’s appearance. In Figure 8a, we put together the P_Tr1_ peak in different Fe–(16–21)%Ga alloys, along with the area under the peak (inset). In order to distinguish the P_Tr1_ effect from total damping, we first fitted P2_Z_ Zener peak by Debye function and then subtracted contribution of thermally activated relaxation from total damping (Figure 8b). It is clear that long-range ordering in alloys with >20%Ga leads to the peak suppressing.

Using positron annihilation spectroscopies and complimentary techniques, Dubov et al. [36] analyzed density of vacancies and linear defects in Fe–(18–21)Ga and concluded that the P_Tr1_ peak is the result of the A2 to D0_3_ transition (with activation energy ~0.4 eV calculated by Kissenger method using DSC results [120]) and decrease in vacancies concentration, which is probably the most powerful structural process at this stage. As a result, Ga-enriched clusters in A2 matrix transforms into partially D0_3_-ordered domain embedded into A2 matrix. Later, this idea about D0_3_-ordered domain embedded into A2 matrix was confirmed and developed using high-resolution neutron diffraction by Balagurov et al. [121,122,123,124].

More recently, an important step forward to better understanding this broad transient effect was done by Fang et al. [58]. They studied influence of alloy composition, heat treatment regimes, and heating rate of TDIF tests on transient peak at about 300–400 °C. Authors denoted this peak as P_wq_, and it corresponds to P_Tr1_ peak in our tests (Figure 8a). They concluded that this transient effect appears only if a sample is quenched from temperatures above the critical temperature of the D0_3_-A2 order–disorder transition, and it disappears if the sample is annealed at 300 °C for 1 h. Additionally, they evaluated activation energy of the P_wq_ peak from experiments with different heating rate by Kissenger method: H = 2.7 ± 0.2 eV, which is close to the activation energy of Ga-Ga dipole pairs relaxation but different to the values of ~0.4 eV calculated by Kissenger method using DSC results [36]. Through deformation studies, they confirmed that dislocations are not the essential source of the P_wq_. The P_wq_ becomes gradually more apparent as the Ga content increases from below ~18 at.%, and the P_wq_ almost disappears if the Ga content reaches 23 at.%.

These experimental observations are also in agreement with the above-mentioned papers, but the conclusion about the mechanism is different. Fang et al. concluded that the potential mechanism of the P_wq_ peak originates from the transition of Ga-rich Guinier-Preston zones to D0_3_ structures or lattice relaxation related to Ga-rich zones, while Golovin et al. explained it by combination of two processes: decrease in thermal vacancy concentration and ordering of rapidly cooled samples [22,36,59]. Nevertheless, both interpretations agree that atomic ordering is involved.

TDIF spectra for several alloys with 16–21%Ga were collected in [59]. Heat treatment influences TDIF curves at heating, and for that reason, TDIF curves at cooling or second heating might be more reliable if we are interested not in transition effects but in thermally activated relaxation.

**Figure 9:**
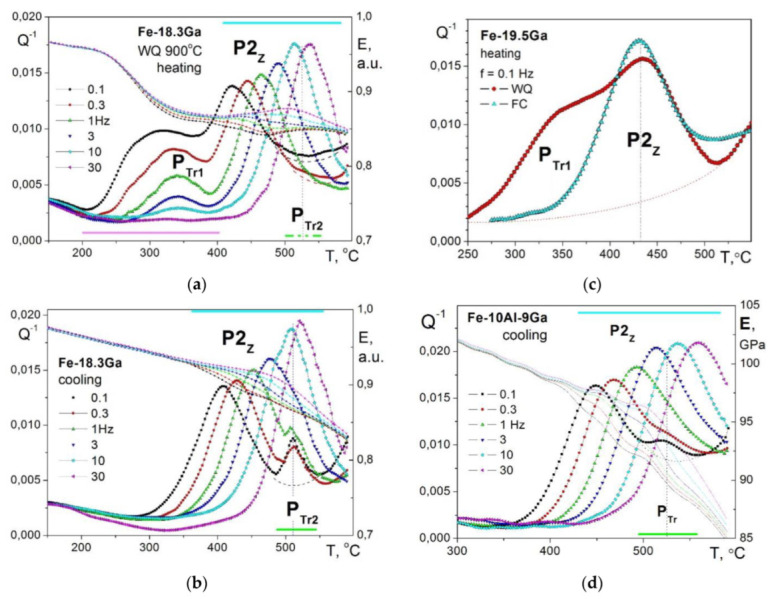
TDIF curves for Fe–18.3Ga after water quenching from 900 °C at heating (**a**) and cooling (**b**); Fe–19.5Ga after water quenching (WQ) and furnace cooling (FC) after annealing at 900 °C (**c**) and Fe–10Al–9Ga (**d**) at cooling.

The P_Tr1_ transient peak observed in the first heating (Figure 9a) of water-quenched sample at ~300 °C (P_Tr1_) disappears at cooling (Figure 9b). It is also absent in a sample after furnace cooling from the same temperature (Figure 9c). In contrast, the transient peak at 510 °C (P_Tr2_) is better distinguished at cooling and in the second heating. Thus, the P_Tr1_ is irreversible, but the P_Tr2_ is reversible effect. Activation energy, H, and average relaxation time distribution, β_τ_ (for f = 1 Hz), which is mainly determined by the distribution in activation energy for the P2 (Zener) peak in alloys with Ga < 23%, are collected in Table 1. Apparent value for characteristic relaxation time is τ_0_ ≈ 10^−18^ s, which is higher than expected values, probably due to some technical problems. The P2 (Zener) and second transient (P_Tr2_) peaks were also recorded for Fe–17.5%Ga single crystal in 3 subsequent heating and cooling runs to 600 °C (only second run was presented in [43]), which excludes any effect of grain boundaries on these effects.

**Figure 10:**
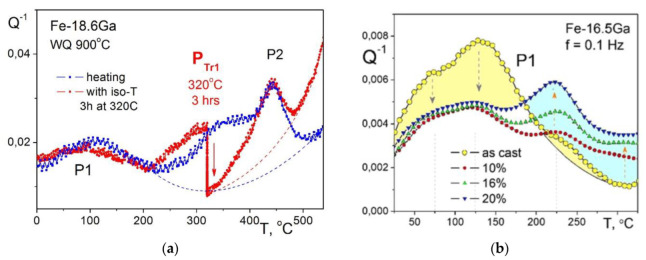
(**a**) Influence of in situ isothermal annealing on the transient peak at 300–350 °C (dotted lines suggest IF behavior in absence of P_Tr1_ peak) and (**b**) influence of cold work on IF below 300 °C.

The paper [59] also added some information for better understanding of the IF peak’s nature. The P_Tr1_ peak also disappears if the measurements are stopped at the peak temperature and IF is measured as a function of time (Figure 10a). P1 is Snoek type, and P2 is Zener peak. In the paper [49], we suggested that there are not enough carbon atoms in our Fe–Ga alloys to produce Snoek-type peak of a given height and proposed that Snoek peak might be overlapped with a peak related to dislocations. Experiments with cold-rolled samples helped to resolve this situation (Figure 10b): cold work leads to appearance of a peak at 200–250 °C but suppresses the P1 peak below 175 °C; this is an additional argument in favor of the Snoek mechanism [59].

**Figure 11:**
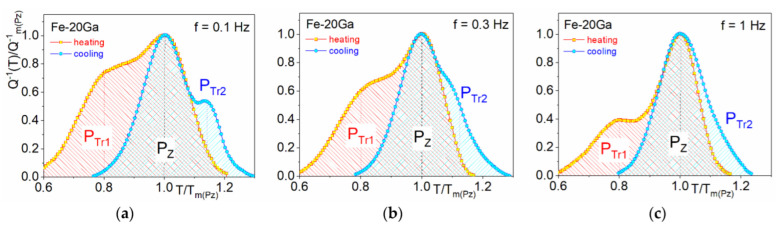
Scheme of the P_Tr1_, P_Z_ (or P2) Zener, and P_Tr2_ peaks combination measured at 0.1 (**a**), 0.3 (**b**), and 1 (**c**) Hz. Peak height and temperature of Zener peak are normalized to 1 on both axes.

Figure 11 presents a scheme (based on experimental data [125]) of interfere between P_Tr1_, P_Z_ (or P2) Zener, and P_Tr1_ peaks for three different frequencies measured at heating and cooling. Central P_Z_ (the Zener) peak can be easily seen if we put together TDIF curves at heating (red color) and cooling (blue color). It has nearly Debye shape, and its temperature increases with increase in measuring frequency. For simplification, the Zener peak height is normalized to “1”. The P_Tr1_ and P_Tr1_ peaks are transient peaks in which height decreases with increase in frequency. This picture helps to understand and predict combination of these peaks at different frequencies.

**Figure 12:**
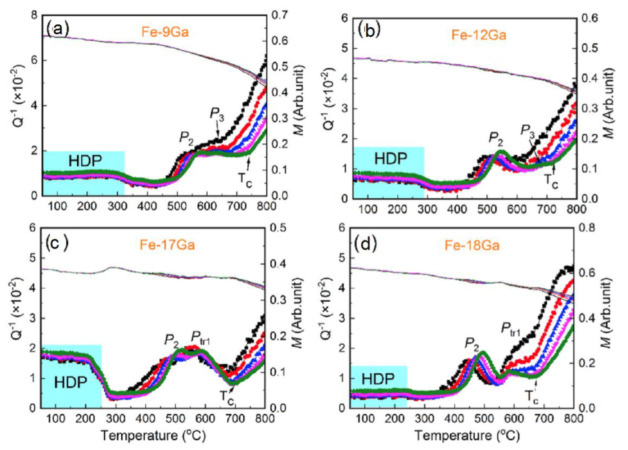
Selected TDIF curves for Fe–Ga alloys measured at torsion [61]. HDP indicates high damping plateau (**a**–**d**).

In agreement with above-discussed results and pioneering publication [4], Sun et al. [61] generalized influence of Ga content in Fe–Ga alloys on so-called high damping plateau (HDP) at room and slightly elevated temperatures and low strain amplitudes of forced torsion vibrations (γ = 3 × 10^−5^) in Fe–(6–18)Ga alloys with A2 structure. Selected TDIF curves from this paper are shown in Figure 12. In their study, 14 days annealed at 480 °C Fe–Ga samples were used: annealing leads to appearance of the equilibrium L1_2_ phase, which significantly decreases damping in HDP in alloys with 18%Ga and more. In as-quenched or as-cast alloys with metastable A2 or A2+D0_3_ structure, the upper concentration limit for HDP is slightly higher.

**Figure 13:**
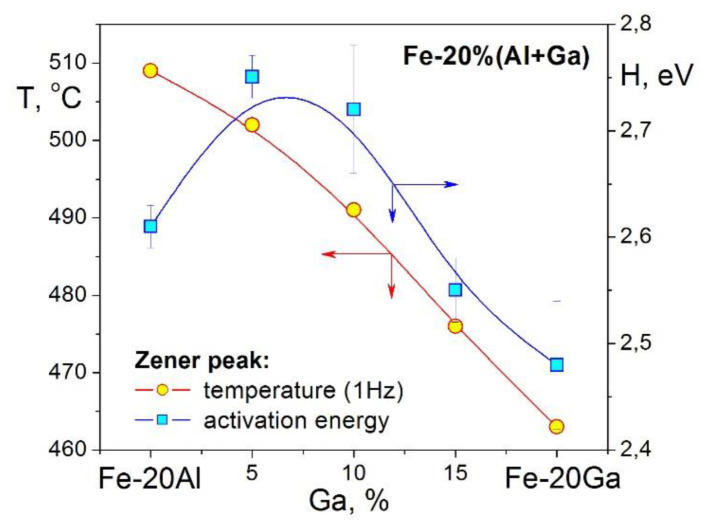
Change of the Zener peak temperature (**left** scale) and activation energy (**right**) in Fe-20%(Al+Ga) alloys.

It should be noted that TDIF spectra for Fe–Ga (Figure 9b) and Fe–Al (Figure 9c) alloys with concentration of substitute atoms below approximately 20–24 at.% are rather similar. In as-quenched states, they exhibit thermally activated Snoek and Zener peaks and transition effects due to ordering (D0_3_)–disordering (A2) transitions. Nevertheless, they should have some quantitative differences. We studied Zener relaxation for several Fe–Al–Ga alloys with total amount of alloying elements about 19–20 at.% [125]. TDIF spectra for Fe–10Al–9Ga alloy at different frequencies are shown in Figure 9d. In Figure 13, information on peak temperature (for 1 Hz) and activation energy (H) is collected. Temperature of the peak increases nearly linearly if Ga atoms are substituted by Al atoms (step in ~5%) in spite of the lattice parameter changes in opposite direction, i.e., decreases from Fe–20Ga to Fe–20Al [19,126]. Relaxation strength decreases nearly linearly if Al is added, while activation energy and distribution of relaxation time pass through extremum. Anelasticity of these alloys is discussed in [64,87].

Damping capacity of Fe–Al–Ga alloys in Hz range is practically independent of the ratio Al/Ga for Fe–18(Al+Ga) [34] and Fe–20(Al+Ga) alloys [64] in contrast with expectations from the Smith and Birchak model [5] and bigger saturation magnetostriction in Fe–Ga alloys. This inconsistency (paradox) was noticed in several above-mentioned and other papers, e.g., [127]. Only once we observed damping as high as Q_h_^−1^ = 0.12 [38] in Fe–Ga once at forced vibrations. However, since it could not be reproduced later, we must consider this result an artifact possibly caused by inner damage of the sample.

Microalloying of Fe–(18–19) Ga alloys with rare earth elements does not significantly change activation parameters of Snoek-type (P1) and Zener (P2) relaxation mechanisms (Table 2) [59].

**Figure 14:**
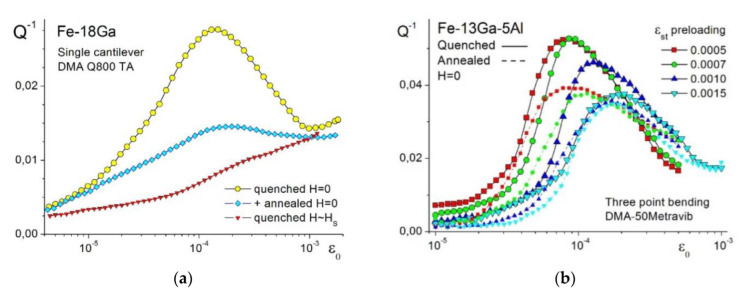
Influence of heat treatment and magnetic field (**a**) and static preloading for as-quenched (solid line, bigger signs) and annealed (dash line, smaller signs) alloys (**b**) on ADIF curves.

**Amplitude-dependent damping** depends strongly on alloy composition, heat treatment, measuring method and temperature, etc. All Fe–Ga-based alloys exhibit a peak on amplitude-dependent internal friction (ADIF) curves in the vicinity of deformation ε = (1–3) × 10^−4^, which disappears if saturated magnetic field is applied (Figure 14a). The magnetic field also suppresses the softening of elastic modulus, proving the magneto-mechanical origin of this effect. The peak height (Q_h.m_^−1^) and consequently the damping capacity (ψ = 2πQ_h.m_^−1^) depend on the alloy composition: Q_h.m_^−1^ at for Fe–18Ga is about 0.05, the compositions with Ga content 21% and 23% exhibit a low damping capacity (~0.01) and becomes higher for Fe–27Ga composition (see Section 3.2).

The influence of heat treatment on damping capacity is rather complicated: for alloys with <13–17%Ga furnace cooling leads to higher damping at room temperature, while residual stresses decrease damping in quenched state. For alloys with about 17–19%Ga quenching leads to higher damping, while furnace cooling and annealing after quenching (Figure 14a,b) decreases damping due to ordering processes or even first-order phase transformation A2 → D0_3_ → L1_2_ in the alloys with 17%Ga and more [34]. In alloys with 21–23%Ga, damping is low even in water-quenched state.

The height of the peak decreases with an increase in test temperature. This decrease has at least two sources: the obvious one is a decrease in magnetization with an increase in testing temperature, and the second one is due to the influence of temperature on the structural state of a sample (this factor is more important for alloys with Ga > 24%).

Damping capacity strongly depends on the measuring method; as a rule of thumb, it is higher if free decay vibrations are used and lower in case of forced vibrations [38]. Three-point bending tests exhibit higher damping values compared to single cantilever tests due to different stress distribution in the same samples [38]. To prove the hypothesis about the influence of clamping force on the samples’ performance, ADIF tests using four different standard screwdriver forces to clamp a sample in the DMA were carried out [86]. Indeed, maximum at ADIF curve (Q_h.m_^−1^) shows a clear decrease with an increase in clamping force: Q_h.max_^−1^ = 0.020 (for screwdriver ‘level 8’), 0.018 (9), 0.016 (11), and 0.0145 (13). This effect is at least partly irreversible: the second test for screwdriver ‘level 8’ gave the value Q_h.m_^−1^ = 0.0165, only. A rise in static loading (static pre-strain from 5 × 10^−4^ to 15 × 10^−4^ for 3-point bending (Figure 14b) or from 2 × 10^−4^ to 20 × 10^−4^ for single cantilever) increases value σ_i_ in Equation (13) and consequently lowers the damping peak as well as shifts the peak position to higher amplitude values [38]. With an increase in static stress, the magnetic domains adjust their shape and volume to the applied stress; consequently, their mobility under cyclic stress becomes lower and damping decreases.

**Figure 15:**
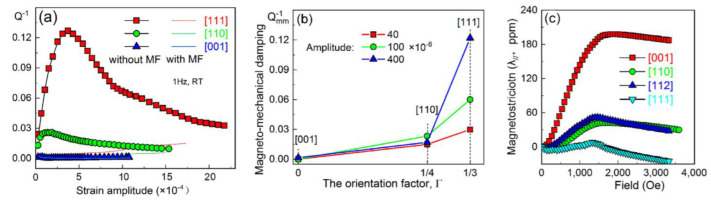
(**a**) the variation of damping capacity with strain amplitude for [001]-, [110]-, and [111]-oriented single crystals at RT at 1 Hz; (**b**) magnetomechanical damping against the orientation factor Γ at strain amplitudes of 0.4 × 10^−4^, 1 × 10^−4^, and 4 × 10^−4^; (**c**) Magnetostrictive curves of [001]-, [110]-, [111]-, and [112]-oriented single crystal samples [56].

The Fe–18Ga single crystals with different orientations were investigated, and anisotropic damping was reported in [56]. The damping of [111]-oriented Fe–Ga single-crystal reaches an unprecedented value of 0.13 (this corresponds to specific damping capacity ψ of ∼0.82), while the damping of [001]-oriented Fe–18Ga single crystal is almost zero (Figure 15a). The domain analysis showed that the magnetomechanical damping originated from irreversible motion at 90°DWs. The damping level correlates well with the orientation factor (Figure 15b) and corresponding magnetostriction (Figure 15c). We also studied damping in the Fe–17.4Ga single crystal [109] at bending and obtained similar values for this orientation (not published).

Even one can often see correlation between maximum damping (Q_h.m_^−1^) and saturation magnetostriction λ_S_ in poly and single crystals as proposed by Smith–Birchak theory (Q_h.m_^−1^~λ_S_) [5]. A better correlation can also be seen with the maximum mobility of domain walls (∆λ/∆H), which is most important for damping of mechanical vibration [127]. The maximal mobility of magnetic domain walls in the applied stress field leads to maximum of damping capacity, followed by further increase in stress Q^−1^~1/σ^2^.

The inconsistency between Smith and Birchak model [5,128] and existing experimental results for α-Fe-based alloys was partly resolved by Sun et al. for Fe–21wt.%Ga–La [54,56]. In ferromagnetic alloys, the damping mechanism is divided into non-magnetic damping (NMD) caused by stress-induced motion of defects, such as point defects, dislocations, grain boundaries, and magneto-mechanical damping (MMD). At low frequency, the MMD caused by the irreversible movement of domain walls (DWs) plays a major role. For the MMD, the distribution of domain configuration and local internal stress barriers are the main factors. The damping according to the Smith and Birchak model is proportional to the saturation magnetostriction (λ_S_) and inversely proportional to the average internal stress (σ_i_), Q_h_^−1^ ∝ λ_S_E/σ_i_, which seems to be strong simplification.

Sun et al. also underlined that although damping is closely related to saturated magnetostriction, there are obvious differences between them [54]. For magnetostrictive applications, it is usually pursued that the restoring force opposing the movement of DWs is as small as possible because it can not only reduce the energy loss but also reduce the critical magnetic field for the saturated magnetostriction. In contrast, for high damping applications, it is necessary to consider the appropriate stress-energy barrier distribution in the matrix because this is a condition for Barkhausen jumps. Therefore, an effective way to obtain high damping is to balance the resistance and mobility of the DWs in the ferromagnetic alloys.

Apart from above-mentioned problems with clamping force, loading scheme, and magnetic domain size, a strong correlation between damping capacity and the coercive force on average magnetic domain size was reported [129]. Beshers et al. proposed a model of Landau’s arrangement of magnetic domains [130] in which the anelastic strain (ε_an_) is described by the change in volume fraction of the closed domains, and stress exerts a force on the 90° domain walls mainly. Sun et al. [54] demonstrated that a normal distribution of internal stress in Fe–21%Ga–xLa alloys better fits experimental data compared with Maxwell’s law. Altogether, it leads to more complex relationship between anelastic strain and magnetostriction.

It is well known that the interaction between second phase and magnetic domain influences the damping capacity of magnetically soft alloys. The total average resistance (σ_ave_) to the movement of the domain in the matrix with particles of a second phase is [54]: σ_ave_ = σ_S_ + (σ_l,a_ + σ_l.d_) + σ_n_, where σ_S_ is the average stress opposing the movement of DWs due to crystalline lattice distortion of the matrix; σ_l,a_ and σ_l,d_ are the resistances related to the increasing area energy of DW and the increasing demagnetization energy induced by the second phase, respectively; and σ_n_ is other forces that hinder the DW movement.

Thus, with the increase in La content in Fe–21Ga–La alloys, the contribution sources of the DWs’ restoring force are gradually transformed from the microstress due to lattice distortion of the matrix to the change of domain wall energy and demagnetization energy related to the Laves phases, in which demagnetization energy gradually becomes the main factor [54]. This implies that the reciprocal pinning effect of the additional and main domains becomes the main hindrance to the domain wall motion in agreement with the experimental results. The large increase in the restoring force also means that higher applied stress is necessary to make the DWs near the second phase move.

**Figure 16:**
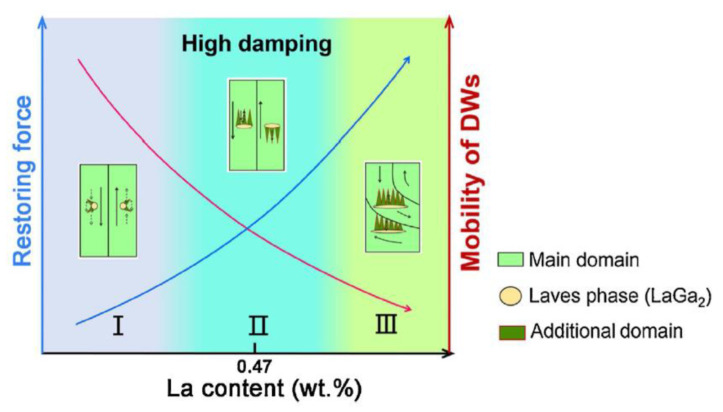
Schematic of the high damping mechanism of Fe–21Ga alloy with proper La doping [54].

In Fe–21Ga–La alloys, the interaction between second phase (the Laves phase) and DW can be divided into three intervals as a function of the La content [54]: first, the average stress related to the lattice distortion of the matrix, which does not significantly change with the increase in La content; the second is the average stress related to the increasing area energy of domain walls, which increases slowly with the increase in La content; and thirdly, the average stress related to the increasing demagnetization energy induced by the Laves phase, which increases rapidly with the increase in La content. With the increase in La content, the resistance opposing DW’s movement increases, but the mobility of the domain decreases. Therefore, proper doping can make the damping of Fe–Ga alloys reach an optimal value (Figure 16).

Another similar innovative idea about increasing damping in Fe–Ga alloys but with higher Ga concentration (about 27%) was proposed by T. Ma’s group: namely, they reported 2–3 times enhanced damping in Fe–(26–27)%Ga by incomplete bcc (A2) to fcc (A1) transition, which in turn produces different structural defects [55]. Our experimental results do not support effectiveness of this method, and we believe that even though this approach helps to increase non-magnetic contribution to damping, it significantly decreases magneto-mechanical component of damping. It is also important to remember that D0_3_ and L1_2_ phases have opposite signs of magnetostriction; as a result, their saturation magnetostriction decreases and may even be equal to zero [46,131,132], which according to the Smith and Birchak approach, leads also to zero magnetomechanical damping. Indeed, most recently published results by another group [63] clearly confirm that two-phase D0_3_ + L1_2_ Fe–27Ga alloy may have different damping mechanisms at lower and higher strain amplitude: D0_3_ phase with higher magnetic damping than non-magnetic damping plays a dominant role under a lower strain amplitude, while L1_2_ phase with higher non-magnetic damping than magnetic damping plays the main role under a higher strain amplitude. Biphase Fe–27Ga alloys with L1_2_ phase have a higher damping capacity only under a higher strain amplitude, which is mainly the non-magnetic damping contribution originating from the twin boundaries movement, but it is not effective at lower amplitude range where magnetomechanical damping plays the main role.

### 3.2. Anelasticity of Fe–Ga Alloys with Ga > 24 at.%

Analysis of TDIF spectra for a wide range of Ga concentration was done in several papers by Golovin et al. for 8–33%Ga [11,49], for 19, 27, and 28%Ga [47,50], and for 23–38%Ga [60]. The important feature of most of these studies is the parallel real-time internal friction and neutron diffraction studies using the same materials with the same heating and cooling rates. This approach to studying anelastic effects is rather new, at least for iron-based alloys, and effective, as it makes the interpretation of anelastic transient effects rather easy as all phase transitions were recorded. Thus, these papers bring both key and detailed information on several transient and thermally activated effects below 600, sometimes below 800 °C. The systematic IF studies for higher temperature range for 0–30%Ga alloys were extended by Meng et al. [61].

In contrast to the alloys with Ga < 27–28%, the structure and phase transitions in the alloys with Ga > 30% were much less studied. Moreover, the study and interpretation of microstructures of Fe–Ga alloys with Ga > 30% are in particular hampered by contradictory phase diagrams: proposed by Kubashewski [15], Köster and Gödecke [14], Okamoto [133], and Bras [134] with respect to so-called M, R, H, and α- and β-Fe_6_Ga_5_ phases presented in these diagrams [11]. Our recent structural studies bring some light to the phase transitions in high-Ga alloys [11,16] and help to interpret anelastic effects in high-Ga alloys. In particular, phase transitions at instant heating for 31–45%Ga alloys were reported using in situ neutron diffraction studies up to 850 °C by Vershinina et al. [26,27,135], which helps to interpret transient effects in the same alloys related to the appearance of the Fe_13_Ga_9_, α- and β-Fe_6_Ga_5_ phases, and to predict new and still unreported anelastic effects. Thus, let us consider Fe–Ga alloys with high Ga content in two steps: alloys below 27–29% and above.

#### 3.2.1. Fe–Ga Alloys with 23 < Ga at.% < 29

Several anelastic effects are recorded in these alloys. Two types of physical mechanisms of the anelastic effects can be clearly distinguished: (i) the thermally activated effects whose temperature increases with an increase in the measuring frequency in agreement with Equation (4) and (ii) transient peaks that accompany phase transitions and whose temperature does not depend on the testing frequency, while the relaxation strength (or peak height) decreases with an increase in frequency in agreement with Equation (13).

**Figure 17:**
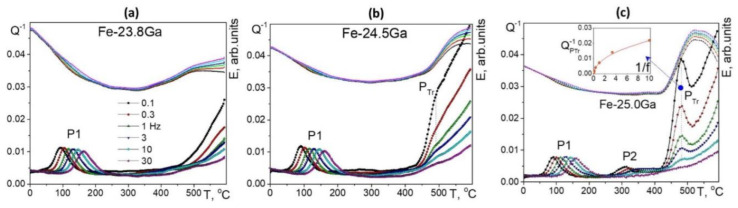
TDIF curves at heating with a rate of 2 °C/min for the Fe–23.8Ga (**a**), Fe–24.5Ga (**b**), and Fe–25.5Ga (**c**) alloys. The dependence of the peak height on inverse frequency for the alloy with 25.5%Ga (inset to (**c**)) [60].

**Figure 18:**
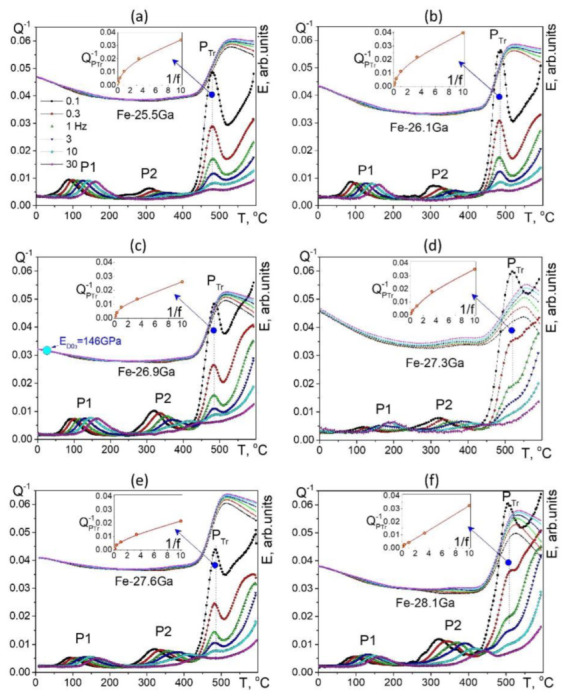
TDIF curves at heating with a rate of 2 °C/min for the Fe–25.5Ga (**a**), Fe–26.1Ga (**b**), Fe–26.9Ga (with absolute value of elastic modulus at RT) (**c**), Fe–27.3Ga (**d**), Fe–27.6Ga (**e**), and Fe28.1Ga (**f**) alloys. Insets: dependences of the peak height on inverse frequency.

Figure 17 (23–25%Ga) and Figure 18 (25.5–28%Ga) give an overview of the TDIF curves below 600 °C measured at forced bending vibrations with 6 frequencies and heating rate of 2 K/min. A total of 2 thermally activated effects, P1 and P2 peaks (Snoek and Zener effects), and a transitory effect at 475–520 °C are typical for these alloys [49,60].

In the as-cast Fe–23.8Ga alloy, the P1 (Snoek-type) peak with Debye-like shape is recorded. A smooth increase in high temperature background above 450 °C is accompanied by an increase in values of modulus (in arbitrary units) above 350 °C. The alloy with 24.5%Ga demonstrates the Snoek-type peak with the same activation parameters, further increase in modulus values, and a weak but clear sign of transient effect at about 475 °C. In agreement with the theory of transient effects, this effect is better pronounced for curves measured at low frequencies. The Zener peak was not recorded in these two alloys due to well-developed ordering. In the alloy with 25%Ga, 2 thermally activated Snoek and Zener peaks and a perfectly pronounced transient P_Tr_ effect at ~480 °C are recorded. The P_Tr_ peak height versus inverse frequency (see Equation (13)) is presented in the inset to Figure 17c.

Figure 17 shows TDIF for 6 alloys with 25.5 to 28.1%Ga. In general, an increase in Ga content in Fe–Ga alloys results in the following effects at TDIF curves:

- Two thermally activated peaks and transient effect, accompanied by sharp increase in modulus;
- The P1 peak height decreases, and the P2 peak height increases. Activation energies (values rounded to tenths) of P1 and P2 peaks are given in Table 3;
- The relaxation strength of the P2 (Zener) peak depends on %Ga in alloys and is discussed below;
- The total height of transient peaks (peak plus background) is approximately constant within accuracy of the measurements 0.05 < Q_Tr_^−1^ < 0.06. High temperature background increases with increase in Ga content leading to non-monotonous height of P_Tr_ vs. Ga content.

Apparent values of activation energy and characteristic relaxation time for P2 effect have undesirable scattering (see Table 3). The P_Tr_ peak temperature increases slightly (25.5%Ga at 480 °C, 26.1%Ga at 485 °C, 26.9%Ga at 487 °C, and 27.3%Ga at 518 °C), which means that the transition needs higher thermodynamic stimulus. A shift in high-temperature background to lower temperatures with increase in Ga content in alloys also takes place as a consequence of a decrease in solidus temperature of the alloys.

**Transient Anelasticity**

**Figure 19:**
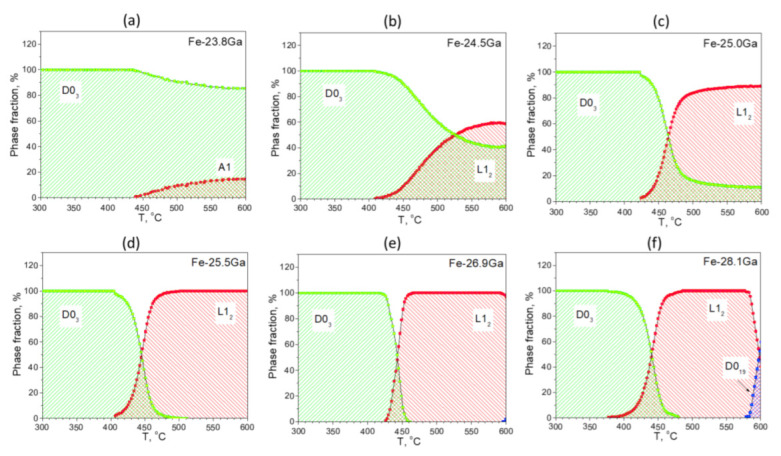
Phase fraction dependencies on the temperature at heating for the Fe–23.8Ga (**a**), Fe–24.5Ga (**b**), Fe–25.0Ga (**c**), Fe–25.5Ga (**d**), Fe–26.9Ga (**e**), and Fe–28.1Ga (**f**) alloys. Dependencies are obtained from the in situ neutron diffraction measurements performed at heating rate 2 °C/min [60].

Let us analyze anelasticity in these alloys starting with the effect of phase transitions. In order to have reliable interpretation of the nature of the internal friction P_Tr_ peak presented in Figure 17 and Figure 18, we have carried out in situ neutron diffraction on the same alloys and with the same heating rate. Simplified and normalized to 100% in situ neutron diffraction (ND) curves for alloys with Ga content from 23.8 to 28.1%Ga are presented in Figure 19 [60].

The D0_3_ to L1_2_ phase transition takes place in all studied alloys. In the alloys from 23.8 to 25.0%Ga, volume fraction of the L1_2_ phase increases, but it is not equal to 100%. For the alloys from 25.5 to 28.1%Ga and temperature range from ~480 to ~600 °C, the L1_2_ phase is the only phase existing in this range, i.e., the D0_3_ → L1_2_ phase transition is 100% completed. These results gave us a background to correct low temperature range of the Fe–Ga phase diagram [16,17].

**Figure 20:**
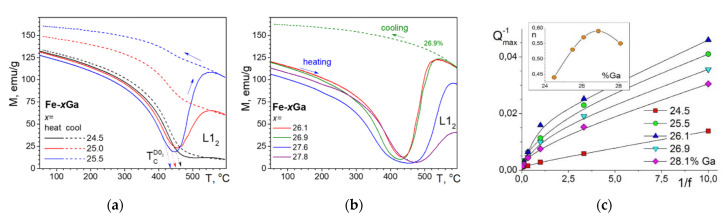
Temperature dependencies of magnetization through heating and cooling with ±6 °C/min rates of Fe–Ga alloys with 24.2–25.5Ga (**a**) and 25.5–28.1Ga (**b**) [60]. The transient P_Tr_ peak height, Q_max_^−1^, as a function of inverse frequency for forced vibrations in Fe–(24–28)Ga (**c**). Inset: values of ‘*n*’ (Q_max_^−1^~1/f*^n^*) as a function of Ga % in the alloys [49].

There is an obvious correlation between internal friction P_Tr_ effect at TDIF curves (Figure 16 and Figure 17) and magnetization curves above 450 °C (Figure 20a,b), which demonstrates a decrease in magnetization of the D0_3_ phase, followed by transition of practically paramagnetic D0_3_ phase to ferromagnetic L_12_ phase. Influence of the frequency of TDIF tests on the P_Tr_ peak height is shown in Figure 20c.

Thus, there is no doubt left that the mechanism of the transient internal friction P_Tr_ peak originates from the first-order transition from metastable D0_3_ to equilibrium L1_2_ phase. Independent of the details of the transition mechanism (discussed in more detail in [21]), the related anelastic strain comes from the lattice deformation when the material is transformed. The D0_3_ → L1_2_ transition orientation relations can be expressed as [136,137]:

(15) $$\begin{array}{c} a_{{L1}_{2}}=a_{{D0}_{3}}\cdot \left(1+\varepsilon_{c}\right)/2,\\ a_{{L1}_{2}}=a_{{D0}_{3}}\cdot \left(1+\varepsilon_{a}\right)/\sqrt{2} \end{array}$$

where the elastic deformation values are estimated to be ε_c_∼0.265 and ε_a_∼0.105.

The presence of the P_Tr_ peak (around 500 °C) depends on the initial state of the alloys. It is always observed at first heating in *as-cast* or *water-quenched* alloys with the D0_3_ structure, and it is absent at cooling or second heating, i.e., after the alloy achieves its equilibrium L1_2_ structure. Additionally, this effect was not recorded after alloy annealing in the D0_19_ range (650 °C, 5 h) followed by water quenching, and it re-appeared after annealing 750 °C for 5 h (B2 range) and quenching [40].

**Figure 21:**
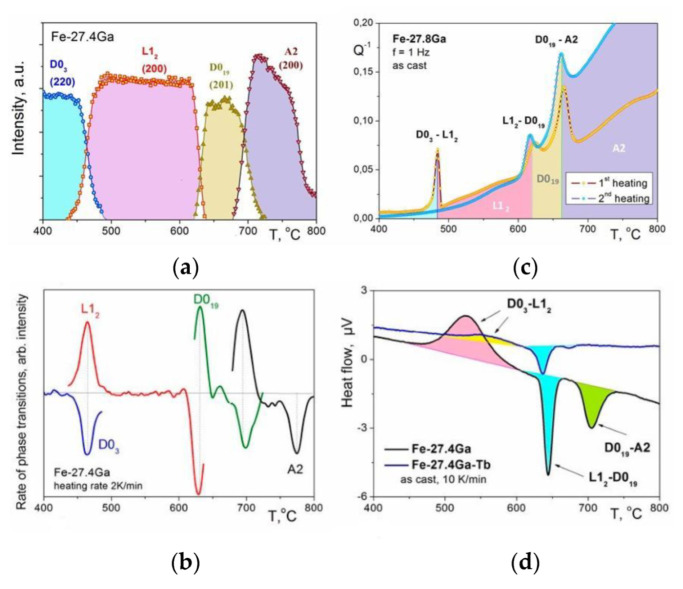

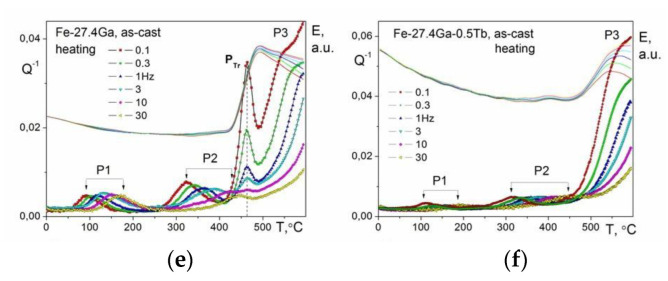
Fe–27Ga type alloys in as-cast state: temperature dependence of (**a**) selected reflections from neutron diffraction for phase identification, (**b**) rate of phase transitions according to neutron diffraction, (**c**) internal friction curves (torsion) with transient peaks due to phase transitions, and (**d**) heat flow (Fe–27Ga and Fe–27Ga–Tb). Heating rate in (**a**,**b**) equals 2 K/min, (**c**)—1 K/min, (**d**)—10 K/min. TDIF and TDEM curves (bending) at different frequencies from 0.1 to 30 Hz for continuous heating (1 K/min) for the parent Fe–27Ga (**e**) and Fe–27.4Ga–0.5Tb (**f**) in as-cast state [46].

At higher temperatures (up to ~800 °C), 2 more transient effects were reported using forced inverted torsion pendulum at EPFL [46,50]. A typical example of several phase transitions in as-cast (or air- or water-quenched) Fe_3_Ga-type alloy is shown in Figure 21 [11,46,47,50]. Three first-order phase transitions (D0_3_ → L1_2_ → D0_19_ → B2) in Fe–(27–28)Ga compositions are accompanied with three transient effects, namely: P_Tr1_ (D0_3_ → L1_2_), P_Tr2_ (L1_2_ → D0_19_), and P_Tr3_ (D0_19_ → B2) [46,47,50]. At this point, researchers should not mix up the P_Tr1_ and P_Tr2_ peaks (Figure 21) for Fe–27%Ga with similarly abbreviated peaks in Figure 6, Figure 7, Figure 8, Figure 9 and Figure 10: they have different natures, discussed in detail in Section 3.1 and Section 3.2, correspondingly.

Figure 21a–c shows details of three first-order phase transitions (D0_3_ ↔ L1_2_ ↔ D0_19_ ↔ A2(B2)) as measured by in situ neutron diffraction. Normalized volume fraction and transition rate as a function of heating rate are shown in Figure 20a,b. To simplify figures, we did not show that the transition from one ordered phase to another one goes through disordering of the ordered phases. For example, the transition from D0_3_ to L1_2_ goes through disordering of the D0_3_ phase to A2, then the transition A2 → A1 takes place, and, finally, the ordering of the A1 lattice leads to formation of the L1_2_ phase [21]. Thus, from the viewpoint of anelasticity, the transition from D0_3_ to L1_2_ structure is practically the same as with bcc (A2) to fcc (A1) transition, and corresponding change in lattice parameter can be described by Equation (14).

The IF peak at about 470–480 °C and an increase in modulus in the first heating of the as-cast sample correspond to the D0_3_ → L1_2_ transition and originate from internal stresses caused by a unit cell volume change due to the D0_3_ → L1_2_ transition and different thermal expansion coefficients of the co-existing phases (D0_3_ and L1_2_ transition). In the Tb-containing alloy, the nucleation and growth of the L1_2_ phases are slower, and the final volume fraction of the close-packed phase is smaller compared with binary alloy [44,45,60]. Consequently, the internal friction transient peak is suppressed in presence of Tb (Figure 21e,f). The mechanism of the terbium influence on the kinetics of the L1_2_ phase growth is as follows: in the Tb-doped Fe–27Ga alloy, a Tb- and Ga-rich phase (about 7–11 at.%Tb) is already formed at casting from a mold state on the grain boundaries of the metastable D0_3_ phase [138,139]. At heating of the as-cast Fe–27Ga alloy, the equilibrium L1_2_ phase nucleates mainly at the grain boundaries of the metastable D0_3_ phase [24,39,40]. In ternary Fe–27Ga–Tb alloy, the grain boundaries are already occupied by the Tb-rich phase, and the L1_2_ phase must find other less favorable places to nucleate and grow.

The anelastic response to the L1_2_ ↔ D0_19_ (P_Tr2_) and D0_19_ ↔ A2(B2) (P_Tr3_) transitions at about 600 and 700 °C is clearly dependent on the measuring frequency and heating/cooling rate [46,47,50]. The D0_19_ → A2(B2) transition depends on heating rate: at 2 K/min, we cannot resolve B2 superlattice reflections, but, at 1 K/min, it becomes possible to speak about B2 ordering of the high temperature A2 phase. All three first-order transitions P_Tr1_ (D0_3_ → L1_2_), P_Tr2_ (L1_2_ → D0_19_), and P_Tr3_ (D0_19_ → B2) are accompanied with corresponding changes (softening) in shear modulus (Figure 20d), which, in the case of the first D0_3_ → L1_2_ transition, is combined with an increase in modulus due to increase in atomic density [46,47,50]. In contrast with the D0_3_ → L1_2_ transition (P_Tr1_), which takes place only at first heating, the T-dot effect can be repeatedly observed in subsequent runs for both the L1_2_ → D0_19_ transition (P_Tr2_) and the D0_19_ → A2 transition (P_Tr3_). On cooling, the A2→ D0_19_ transition is almost undetectable, and D0_19_ → L1_2_ is much more spread out over temperature. It is likely that part of the transformation at cooling occurs directly from A2 to L1_2_ in agreement with neutron diffraction observations [20].

The P_Tr1_ (D0_3_ → L1_2_) and P_Tr3_ (D0_19_ → B2) transitions are characterized by change in sample atomic density (68% ↔ 74%) and atomic volume per atom in unit cell. In contrast, the P_Tr2_ (L1_2_ → D0_19_) transition is characterized by ferro- (L1_2_) to paramagnetic (D0_19_) transition and the narrow range of coexistence of these two phases (595–606 °C [18]) but not by change in atomic packing density which is 74% for both phases. Most probably, the P_Tr2_ peak for the L1_2_ → D0_19_ (or fcc → hcp) phase transition is due to a long-range motion of Shockley dislocations, which assists the transition between these two close-packed phases.

D. Mari et al. [50] fitted the transient peaks with a Lorentzian function to obtain the peak height as a function of heating rate. The change was not linear. For the peak P_Tr2_ (L1_2_ → D0_19_),

(16) $$A_{P2}=C_{1}\cdot {\left(\dot{T}\right)}^{0.18}$$

and the peak P_Tr3_ (D0_19_ → A2/B2):

(17) $$A_{P3}=C_{2}\cdot {\left(\dot{T}\right)}^{0.29}.$$

All the transient peak heights vary with the measuring frequency; for the P_Tr2_ peak,

(18) $$A_{P2}=C_{1}\cdot {\left(\omega \right)}^{-0.22}$$

and the P_Tr3_:

(19) $$A_{P3}=C_{2}\cdot {\left(\omega \right)}^{-0.44}.$$

The ‘*n*’ values for frequency dependence of the transient effects for alloys with 13–38%Ga were also studied using bending vibrations at DMA Q800 below 600 °C in several papers [33,49,59,60]. To summarize, the relaxation strength of all transient peaks in binary Fe–Ga alloys related to the first- or second-order phase transitions is proportional to heating rate (Ṫ) and inverse frequency with a certain power dependence, i.e., with a coefficient to the power of *n* (Q_Tr_^−1^~Ṫ/f*^n^*, where f = ω/2π). We never observed inverse linear but always power proportionality between IF and inverse frequency.

Table 4 presents values of coefficient *n* for the first-order D0_3_ to L1_2_ and D0_3_ to Fe_13_Ga_9_ transitions (this paper and [49]) and for the second-order D0_3_ to A2 transition [59] to illustrate a wide range of Ga concentrations in binary Fe–Ga alloys. This coefficient is about 0.9–0.7 for the second-order transition (D0_3_ to A2) in the alloys with 16.5–19.5%Ga.

This parameter is in the range of 0.5–0.7 for the alloys with 25.5–28.1%Ga with the first-order transition from D0_3_ to L1_2_. This transient effect originates from a change in the atomic volume of coexisting phases and a corresponding rise of the local stresses and strains due to change in the ratio between bcc- and fcc-born phases (D0_3_ and L1_2_). Microalloying of Fe–Ga alloys by RE (Tb, Yb, Er, Sm, Pr, La, and Dy) decreases or does not influence this transient effect, as these elements were found to have similar influence on the rate of the transition [11,16,17,44,45,59,60].

Finally, the values of *n* due to more complicated, maybe two-stage transition in the alloys with 28.9–38.4Ga (D0_3_(B2) + Fe_13_Ga_5_ to L1_2_ + Fe_6_Ga_5_) are rather scattered from 0.7 to 0.2, as discussed in the next section.

As of yet, most IF studies were carried out for the first out of three transitions reported in Table 5 as it has practical importance for as-cast (or as-quenched) samples. The results for high Ga > 30% alloys need additional verification in the future.

**Thermally Activated Anelastic Effects**

Apart from transient anelastic effects, two well-shaped thermally activated effects are recorded in Fe–(23–28)%Ga, as seen in peaks P1 and P2 in Figure 17 and Figure 18. A lot of studies were carried out to interpret these peaks [10,11,33,36,37,38,39,40,41,42,43,46,47,49,50,60]. For that reason, we summarize here the results in a relatively short way: the main hypothesis is that the P1 is the Snoek-type peak and P2 is the Zener peak in Fe–Ga and Fe–Ga–Al alloys.

Before providing several valuable arguments in favor of these interpretations, we would like to mention a couple of counterarguments to be not forgotten. The main problem with the interpretation of the P1 peak as a Snoek-type peak, in spite of its reasonable activation parameters, is its height. As discussed in [49], the reported height of the peaks (Q_m_^−1^ up to 0.08) can be achieved in the alloys with carbon content of roughly 0.08 wt.%. From the alloys’ composition, the carbon content in the used alloys is lower, and overlapping two relaxation mechanisms point defect (Snoek-type) and dislocation-related relaxation was proposed in [49]. Concerning the P2 (Zener) effect, it is important to remember that this effect depends greatly on both second-order transition (i.e., it depends on alloy composition with respect to A3B and AB ordering and temperature of measurements with respect to order–disorder transition) and first-order transition (i.e., on the D0_3_ to L1_2_ transition in studied alloys).

**Figure 22:**
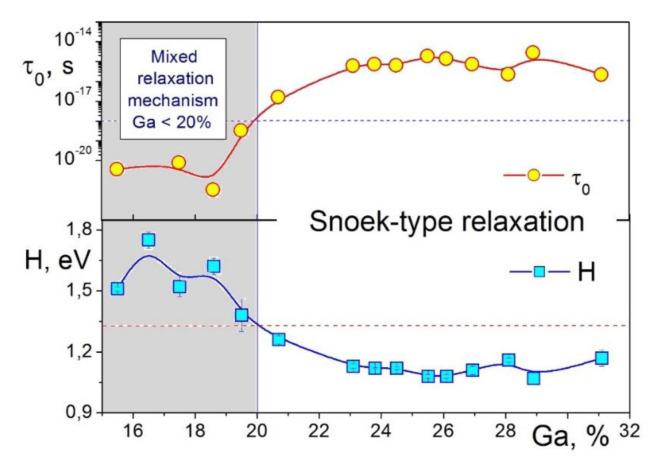
The relaxation time (upper) and the activation energy (lower raw) for the P1 thermally activated peak as a function of Ga content in binary Fe–Ga alloys.

**The P1 Snoek-type effect** (Figure 17 and Figure 18) is well recorded in the alloys with Ga content up to 31%. The P1 peak in the D0_3_-ordered alloys with 20–28%Ga has a clear Debye-like shape, and it is undoubtedly a thermally activated effect. The P1 effect is rather asymmetrical in disordered alloys with 9–19%Ga due to overlapping with another transient and thermally activated effects. The apparent values of activation energy and pre-exponential factor of relaxation time are summarized in Figure 22.

The values of *_0_* between 10^−13^ and 10^−18^ s can be, to some extent, assigned to the point defects relaxation (Snoek type mechanism). This interval is broader than the classical range predicted by theory for the point defects [96] due to technical uncertainties in the recording temperature inside relatively massive samples compared with the temperature measured by a thermocouple. The relaxation time, which is faster than 10^−18^ s, can be hardly explained by a single relaxation process, and, most probably, these values are the result of overlapping of a thermally activated P1 peak with some reorganization of the structure discussed above (vacancy annihilation, and ordering–disordering [43,44]). Thus, we called this frequency range a ‘dark area’ and excluded the results from this area from the discussion below. The activation energies of the P1 peaks with the relaxation time *τ_0_* < 10^−16^ s are roughly 1.1 eV (Table 2). These values are rather close to those for Fe–Al alloys with similar concentration of substitute atoms [66].

In agreement with earlier papers [30,33,37,67], the atomic ordering in the Fe–Ga alloys with Ga > 20% leads to the peak narrowing and a related increase in the peak height. The main argument in favor of the Snoek-type origin for the nature of the P1 effect is that the activation parameters (apparent activation energy and characteristic relaxation time) for this anelastic effect are similar to those for the Snoek-type relaxation in several bcc iron-based alloys with not-(strong)-carbide forming elements: Fe–Al, Fe–Si, Fe–Co, and Fe–Ge [68,140]. The same hypothesis is also given in several papers from other groups without additional evidence of the mechanism.

As mentioned above, the ratio between peak height and carbon content is a critical issue. All the studied alloys were produced similarly from the same raw materials (99.99% Fe and 99.99%Ga purity). The Snoek peak height in the studied alloys, Q_m_^−1^ ≤ 0.007 requires about 0.02 at.% C in the solid solution. This amount of carbon is higher than the carbon content (<0.007 at.% C) in the alloys, according to their chemical analysis. Thus, to explain the P1 peak, especially the asymmetrical one in the alloys with Ga < 20%, we propose two additional acting mechanisms which might overlap with the Snoek-type relaxation to form the P1 effect. They are as follows:

(1) The P1 effect can be caused, at least partly, by the γ relaxation. This relaxation effect is due to the kink pair formation on screw dislocations in bcc metals or alloys with the activation energy H_γ_ = 2H_K(screw)_ + H_M(screw)_ − 2k_B_T, where H_K(screw)_ and H_M(screw)_ are the energies of formation and motion of a single kink on screw dislocation, correspondingly [111]. The main disagreement of the experimental results with the existing theory of the γ-relaxation theory [111,141,142] is in extremely fast values of the characteristic relaxation time (Table 3). The apparent experimental values fit better to the point defect relaxation, i.e., to the Snoek-type effect, while the dislocation related relaxation has slower relaxation time;
(2) The P1 effect can be also caused by reorientation of pairs of vacancies under applied cyclic stress. Similar ideas were proposed for Fe–Al [143] and Fe–Cr alloys [144]. The arguments in favor of this hypothesis are a decrease in the P1 peak effect after annealing, which leads to a drastic decrease in vacancy concentration in Fe–Ga alloys [22], and the experimental values of characteristic relaxation times, which are in favor of the point defect relaxation.

None of these hypotheses is fully proved at this stage. Further arguments in favor of one of these hypotheses should be obtained after further research.

After quenching from 650 °C, i.e., from the hexagonal D0_19_ range of the phase diagram, or after first heating of a water-quenched sample to 600 °C (the structure corresponds to the L1_2_ phase), the P1 peak was never recorded. This fact fully agrees with the main statement of the Snoek theory, which is applicable for the bcc lattices only, and consequently, the Snoek-type relaxation was found in the samples neither with the hexagonal D0_19_ nor with the fcc-based L1_2_ structures supporting the interpretation that the Snoek mechanism is the main mechanism for the P1 effect in Fe–Ga alloys. An interesting observation on thermally activated Snoek and Zener effects in Fe–27Ga alloy is added by Li et al. [62]: with increasing proportion of L1_2_ phase, the peak position of Snoek-type relaxation peak (*P*1) remains stable, and the height decreases and finally disappears, while the height of Zener-type relaxation peak (*P*2) decreases gradually, and its peak position moves toward higher temperatures. They also confirm conclusions [131] that the domain rotation or domain wall movement of L1_2_ phase needs higher magnetic fields or higher stress than those for bcc phases [62].

It is notable that the P1 peak in Fe–Ga–Al alloys [37,125] sometimes has two very clear heads, which can be easily explained by carbon atom jumps in the positions Fe–C–Al and Fe–C–Ga, similar to those in Fe–Al–Si alloys [80,81]. Microalloying of Fe–Ga alloys by RE (Tb, Yb, Er, Sm, Pr, La, and Dy) does not influence the activation parameters of the P1 effect much but may decrease the peak height (Tb, Sm, and Pr) [60].

**The P2 (Zener) effects** in Fe–Ga alloys with Ga < 25 at.% are also undoubtedly thermally activated effects with the activation parameters collected in Table 3 for the TDIF tests performed both at heating and cooling. The nature of this peak, in agreement with its activation energies and relaxation times, is stress-induced diffusion-controlled reorientation of the Ga-Ga atom pair in Fe–Ga solid solution. Importantly, the relaxation strength of the Zener relaxation depends not only on the amount of Ga atoms but also on their ordering, i.e., on A2 to D0_3_ transition, and first-order D0_3_ to L1_2_ transition.

**Figure 23:**
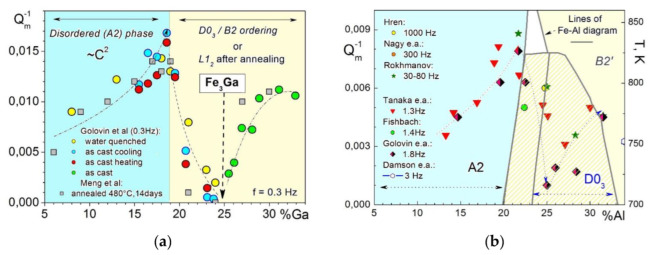
Dependence of the P2 Zener peak height on Ga content for alloys in metastable state (as quenched to as cast for forced bending vibrations) by Golovin [49] and in nearly equilibrium state (annealed at 480 °C for 14 days for forced torsion vibrations) by Meng et al. [61] (**a**). The same dependence for Fe–Al system for free-decay torsion and bending vibrations adapted from [66,68,138] (**b**).

The relaxation strength for the Zener relaxation (Δ_Z_ = 2Q_m_^−1^) depends on the Ga concentration in the binary as-quenched and as-cast Fe–Ga alloys (circles in Figure 23). An increase in the Zener peak height, Q_m_^−1^, with an increase in the Ga content up to 18–19 at.%, i.e., in the A2 structures, is followed by a rapid decrease in the peak height for the alloys with >19%Ga, i.e., in the ordered D0_3_ structure, and the peak practically vanishes at stoichiometric composition Fe_3_Ga [49]. Similar observations were confirmed by Meng et al. [61] for Fe–Ga samples annealed at 480 °C for 14 days. Similar results were reported: the same increase in the peak height in A2 range while the rapid decrease in the peak above 19%Ga was due to formation of the ordered L1_2_ phase. We added their results to Figure 23 as grey squares (values may not be very precise as we picked them up from the figures in paper [61] “by eye“). It should be noted that the TDIF curves at cooling from 600 °C (the D0_3_ to L1_2_ transition is completed at heating) do not show the Zener peak, and it was concluded that in strongly ordered equilibrium L1_2_ phase the Zener peak is absent due to ordering effect [49,60]. If this viewpoint is accepted, the results published in Ref. [61] can be interpreted in terms of Zener peak in D0_3_ phase of two phase D0_3_ + L1_2_ structure of the samples.

We have already mentioned that Fe–Al (equilibrium) and Fe–Ga (metastable) diagrams are rather similar. It is not surprising that Zener relaxation in both systems expresses similar dependence on concentration of substitute element and ordering: this is well seen if we compare the results in Figure 23a,b.

The relaxation strength for the Zener relaxation depends on the concentration of substitute atoms and the degree of order, i.e., on the order parameter (η). The ordering degree increases with Ga % (or Al %) in the alloys and reaches maximum at Fe_3_Ga composition. In agreement with Equation (13), an increase in the Ga concentration in the Fe–Ga alloys up to ~19 at.%, i.e., in the A2 range until the value of the parameter f(χ_o_,C_Ga_) ≈ 1, leads to an increase in the Zener peak height. At higher Ga content, the effect of ordering noticed by neutron diffractions plays a more powerful role as compared with the effect of concentration, and the Zener peak height decreases rapidly.

**Figure 24:**
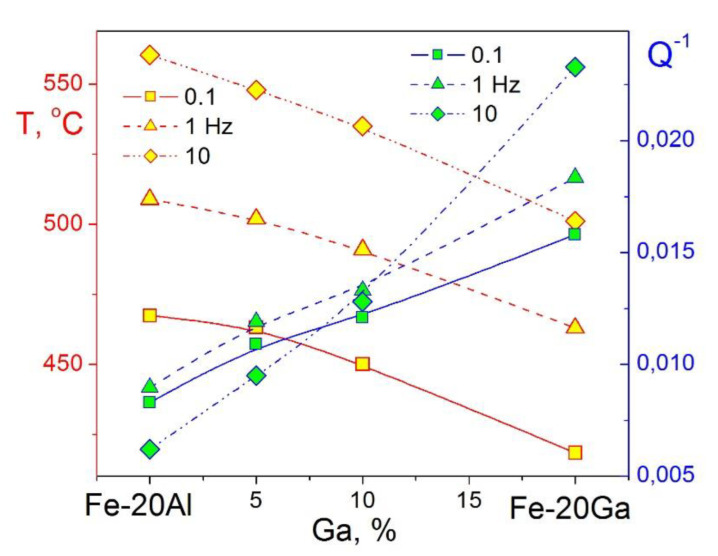
Zener peak temperature and height in Fe–20%(Al+Ga) alloys for measurements at three different frequencies.

One can also see that relaxation strength of Zener relaxation (Δ = 2Q^−1^) is different in these two systems. Figure 24 shows a decrease in the Zener peak temperature and an increase in the peak height with step-by-step substitute of Al by Ga atoms in Fe–20%(Al+Ga) alloys measured at three different frequencies: analysis of Zener relaxation in Fe–Al–Ga ternary alloys suggests that the total effect is composed from contribution of three types of atomic pairs, Al-Al, Al-Ga, and Ga-Ga [125].

**Figure 25:**
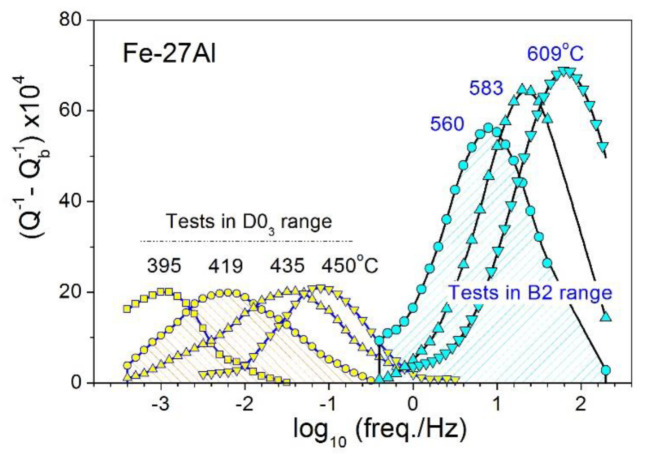
Frequency-dependent internal friction after background subtraction for Fe–27Al alloy measured at fixed temperatures during step-by-step increase in temperature in order to correspond to the ranges of D0_3_ and B2 phases [86].

Usage of FDIF tests to study Zener relaxation mechanism, as demonstrated for Fe–27Al alloy in [86], may provide some additional information which is not possible to obtain in TDIF tests for two reasons. First, the Zener peak is influenced by the D0_3_ ↔ B2 transition (see Figure 23b); second, the same transition during TDIF tests changes parameters of Zener relaxation in the D0_3_ and B2 structures directly during measurements. The Zener peak parameters depend on ordering–disordering of the Fe–Al alloys and, therefore, should be different in these phases. This effect is discussed in detail for Fe–(22–28)Al alloys, and activation energies are compared with results of the tracer diffusion experiments [72,75]. Indeed, it is obvious that the height of the Zener peak is different if it is measured in the D0_3_ or the B2 temperature range (Figure 25) [86]: B2 structure corresponds to concentration of Fe and Al of 1:1, in contrast with D0_3_ phase with ration 3:1 and, therefore, is roughly twice less ordered in the Fe–27Al alloy, resulting in higher Zener peak.

The peak height increases with a step-by-step increase in the temperature of the frequency-dependent internal friction tests and a corresponding decrease in the degree of ordering. Using the Arrhenius plot to treat the TDIF results without distinguishing between the points belonging to D0_3_ and B2 phases introduces errors in the calculations. Consequently, the isothermal mechanical spectroscopy frequency-dependent tests [72,75,86] demonstrate the difference in the activation energy of the Zener relaxation in D0_3_- or B2-ordered states in the Fe–27Al alloy: 2.97 ± 0.08 eV for D0_3_ or 2.44 ± 0.02 eV for B2 state. With an increase in the order parameter (η) in the Fe–Al alloys, the activation energy of self-diffusion increases in agreement with Girifalco’s theory [145]: H(η) = H_η=0_ (1 + αη^2^), where α is a structural parameter. The activation energies of the Zener relaxation in Fe–26Al alloy and diffusion [146] in the B2 and D0_3_ phases have the same sequence, i.e., H_B2_ < H_D03_. The activation energies of the Zener relaxation are only slightly lower than those of inter-diffusion in B2 [132], practically the same as those of the Al tracer self-diffusion in B2 [147], and slightly higher than that of the Fe tracer self-diffusion in the B2 and D0_3_ [146]. This proves mechanical spectroscopy to be a useful tool for a relatively quick estimation of the activation energies for atoms diffusion in a substitutional solid solution in different ranges of the phase diagram.

Similar experiments for Fe–27Ga alloys might be particularly interesting as it is possible to measure parameters of the Zener relaxation in three different phases: D0_3_, L1_2_, and D0_19_ on the same sample by playing with the temperature of isothermal FDIF tests and frequency range. As of yet, such experiments have not been carried out. Some information on Zener effect in ternary Fe–20(Al+Ga) alloys is presented in Figure 11, Figure 13, and Figure 23.

#### 3.2.2. Fe–Ga Alloys Ga > 30 at.%

These alloys are less studied. The main reason is that they are less attractive for practical application due to low magnetostriction, brittleness, and low melting points. For the same reason, their structure was not studied in detail. Most recently, some efforts to study high gallium Fe–Ga alloys structure (up to 45%Ga) and anelastic effects (up to 38%Ga) were conducted. Quasi equilibrium structure of the alloys after 300 h [16] and longer annealing time [17] below 600 °C was reported, and it was shown that even 1800 h of annealing does not allow reaching 100% equilibrium structure due to sluggish diffusion. Phase transitions at instant heating and cooling with the rate ±2 K/min were reported by real-time diffraction experiments for Fe–45%Ga [26], Fe–38%Ga [135], and 31–35%Ga [26,27,135]. Corresponding TDIF tests with the same heating rate were carried out [60].

**Figure 26:**
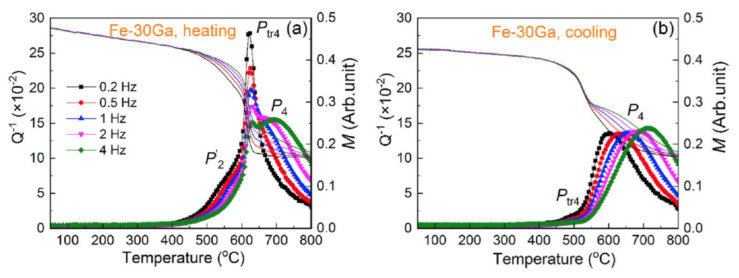
TDIF curves and relative modulus–temperature curves in continuous heating (**a**) and cooling (**b**) processes for 480 °C; 14 days annealed Fe–30Ga sample at 5 different frequencies of 0.2, 0.5, 1, 2, and 4 Hz [61].

A transient effect above 600 °C (torsion forced vibration) was reported for the Fe–30Ga alloy (Figure 26a); it is explained by the L1_2_→A2/B2 phase transition according to [61]. On the other hand, the equilibrium phase diagram suggests [15] that the L1_2_ → D0_19_ + Fe_6_Ga_5_ reaction takes place at 619–625 °C, followed by the Fe_6_Ga_5_ → B2 transition between 625 and 653 °C. Nevertheless, taking into account a sluggish diffusion and transformation rate, the simplified interpretation of this transition as L1_2_ → B2 can also be accepted. The grain boundary peak at cooling is recorded between 700 and 600 °C with the activation parameters (Figure 26b): H = 2.08 eV and τ_0_ = 10^−12^ s [61]. From asymmetry of this peak and modulus increase below 550 °C, it is possible to suspect that the above activation parameters might be influenced by the phase transition from high temperature phases mentioned above to the mixture of low temperature equilibrium phases (L1_2_ + α- or β-modification of Fe_6_Ga_5_; transition from β → α modification is also slow process).

**Figure 27:**
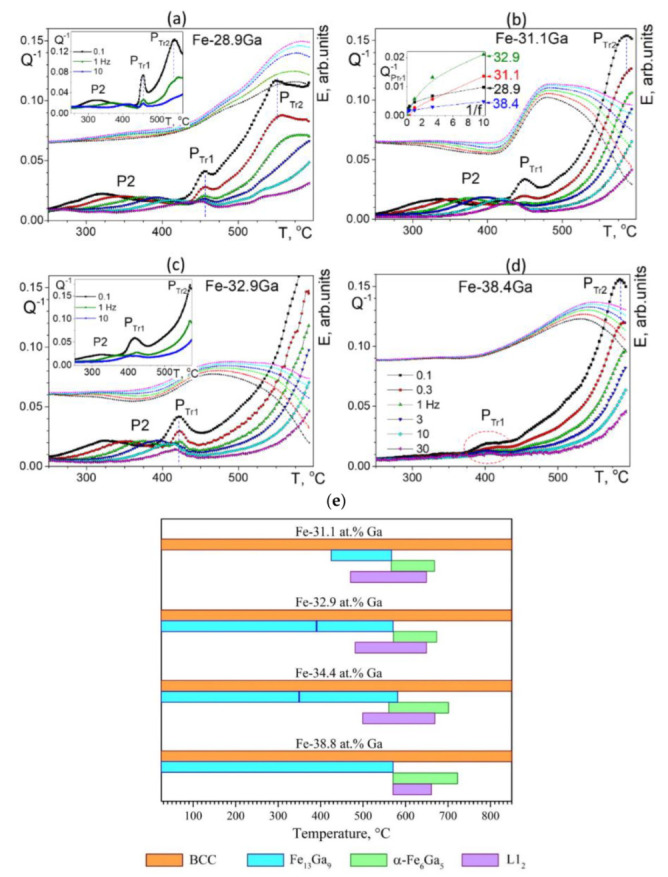
(**a**–**d**) TDIF curves at heating with K/min rate for the Fe–28.9Ga (**a**), Fe–31.1Ga (**b**), Fe–32.9Ga (**c**), and Fe–38.4Ga (**d**) (insets to (**a**,**c**): TDIF curves for the water-quenched samples from 800 °C for the alloys Fe–28.9Ga and Fe–32.9Ga. Inset to (**b**): the P_Tr1_ peak height as a function of inverse frequency in Fe–(29–38)Ga); temperature ranges for the existence of phases during heating up to 850 °C (**e**). The vertical segments indicate the temperatures corresponding to significant increase in the volume fraction of Fe_13_Ga_9_ upon heating [27].

With further increase in Ga content up to 38.4% (we were not able to test Fe–45%Ga sample because of its brittleness), the TDIF spectra of as-cast Fe–Ga alloys change and become more complicated due to new phases and phase transitions (Figure 27). The P1 peak vanishes smoothly; it becomes smaller and smaller in the alloys with 28.9 and 31.1%Ga, and it is undistinguishable in the alloys with 32.9 and 38.4%Ga due to decrease in volume fraction of bcc-derived phases. Thus, the TDIF curves in Figure 27 start from temperature of 250 °C to better show the P2 peak and transient effect. Activation parameters for the P2 peak are: 28.9%Ga: H = 1.61 ± 0.08 eV, τ_0_ = 3 × 10^−14^ s, 31.1%Ga: H = 1.43 ± 0.08 eV, τ_0_ = 1 × 10^−16^ s, 32.9%Ga: H = 2.0 ± 0.3 eV, τ_0_ = 1 × 10^−17^ s [60].

The transient effect (denoted as P_Tr1_ in Figure 27) earlier assigned to the D0_3_ to L1_2_ transition moves to lower temperatures (from about 500 °C in alloys with 27–28%Ga to 400 °C in alloy with 38%Ga) and decreases. The reason is the presence of the Fe_13_Ga_9_ phase in the structure of the alloys with 32–38%Ga already at room temperature [27], which leads to the corresponding decrease in bcc-derived phases (D0_3_, B2) and their transition to the L1_2_ phase and to a pronounced increase in a volume fraction of the Fe_13_Ga_9_ phase above 400 °C which, most probably, contributes to the change of P_Tr1_ effect temperature and parameters. Structure of the alloy with 38.4%Ga in as-cast state is the mixture of the Fe_13_Ga_9_ phase (main phase) and B2. Between 500 and 600 °C (P_Tr2_ effect) the L1_2_ and α-Fe_6_Ga_5_ structures appear, and the metastable Fe_13_Ga_9_ phase disappears (Table 6).

The P_Tr1_ peak can be additionally enhanced by quenching of as-cast 28.9 and 32.9%Ga alloys from 800 °C in cold water (insets to Figure 26a,c). Inset to Figure 27b shows dependence of the P_Tr1_ peak height against inverse frequency, which is rather similar to the comparable dependencies for alloys with lower Ga content. The P_Tr2_ peak is clearly seen in the alloys with 28.9Ga, Fe–31.1Ga, and Fe–38.4Ga. More systematic measurements in this concentration and temperature range might be helpful.

**Figure 28:**
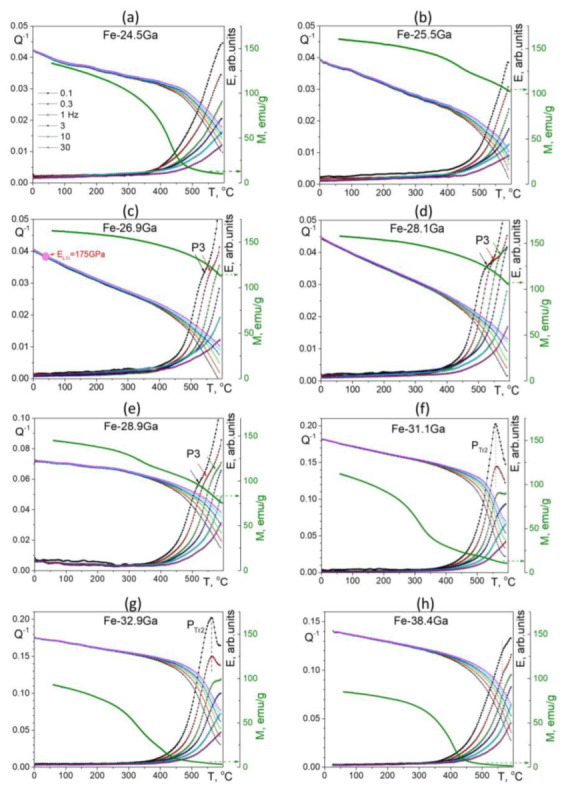
Q^−1^(T), E(T) (cooling rate 2 °C /min) and M(T) (cooling rate 6 °C/min) curves at cooling for the Fe–24.5Ga (**a**), Fe–25.5Ga (**b**), Fe–26.9Ga (with absolute value of elastic modulus at RT) (**c**), Fe–28.2Ga (**d**), Fe–28.9Ga (**e**), Fe–31.1Ga (**f**), Fe–32.9Ga (**g**), and Fe–38.4Ga (**h**).

Temperature-dependent IF curves for Fe–(24–38)Ga at cooling from 600 °C are rather similar to each other, and they are very different compared to those recorded at heating (Figure 28). TDIF at cooling does not have either P1 and P2 peaks or P_Tr_ peaks [60]. The reason is simple: during heating to 600 °C, most studied alloys get either 100% or significant amount of the L1_2_ phase. This phase is ordered fcc phase, and the amount of equilibrium L1_2_ phase below 600 °C does not significantly change at cooling. Absence of any sharp transition explains the absence of P_Tr_ peak at TDIF curves. Presence of L1_2_ phase explains higher magnetization of the samples up to room temperature. The P1 peak, as a Snoek-type peak, does not exist in other than bcc lattice, which is also true for fcc-derived L1_2_ structure. The P2 peak assigned to the Zener relaxation is also suppressed in well-ordered L1_2_ structure.

TDIF curves at cooling for alloys with Ga content below 26.9% consist of low temperature (below approximately 400 °C) and high temperature background (Figure 28). With an increase in Ga content up to 27–29%Ga, a new thermally activated peak (denoted as “P3”) appears at the high temperature background. Very rough estimation of activation parameters for these peaks at cooling can be given based on 2–3 experimental curves for 27%Ga (Figure 21) and 28–29%Ga (Figure 28): H = 2.5–3.0 eV. In agreement with the FDIF results reported by isothermal mechanical spectroscopy [30] and TDIF tests at forced vibration torsion pendulum [46,61], one can suggest that the P3 peak can be a grain boundary peak measured by DMA below 600 °C. Second run of these samples is similar to cooling curve underlying the fact that further heating and cooling between RT and 600 °C does not change equilibrium L1_2_ structure of the samples. Further increase in Ga concentration to 31–33%Ga leads to formation of a (P_Tr2_) transient effect at cooling. This transient peak can possibly mask the P3 peak.

## 4. Summary

To summarize, we provided information on several thermally activated relaxation effects in binary Fe–Ga alloys: in detail on the Snoek-type (in most figures denoted as the P1 with an activation energy of about 1.1 eV) effect and the Zener (in most figures denoted as the P1 with activation energy decreasing with increase in Ga concentration roughly from 2.5 to 1.8 eV) effect, and in brief about low-temperature dislocation-related peaks and grain boundary peak. A cascade of transient effects was discussed: the structural (P_Tr1_) transition at about 300–400 °C in metastable alloys with Ga < 20%, the reversible P_Tr2_ effect due to D0_3_ ↔ A2 second-order phase transition also for the alloys with Ga < 20%, the irreversible P_Tr_ (also denoted as P_Tr1_ at some figures) due to the first-order D0_3_ → L1_2_ phase transition at 450–500 °C in Fe–(24–33)Ga, the reversible P_Tr2_ and P_Tr3_ peaks due to the L1_2_ ↔ D0_19_ (600–620 °C) and D0_19_ ↔ B2 (670–700 °C) transition in Fe–(27–28)Ga, and the transient effects at 400–600 °C from alloys with Ga > 30% due to a change from D0_3_ + Fe_13_Ga_9_ to L1_2_ + Fe_6_Ga_5_ phases. In all cases, the relaxation strength of all transient peaks in binary Fe–Ga alloys related to the first- and second-order phase transitions is proportional to the heating rate (Ṫ) and inverse frequency with a certain power dependence, i.e., with different coefficients to the power of n (Q_Tr_^−1^~Ṫ/f^n^). Amplitude-dependent effects in Fe–Ga alloys were also observed and analyzed with less success: in contrast to the expected high damping capacity, the Fe–Ga polycrystalline alloys do not exhibit high amplitude-dependent magneto-mechanical damping in spite of their high saturation magnetostriction.

**Figure 29:**
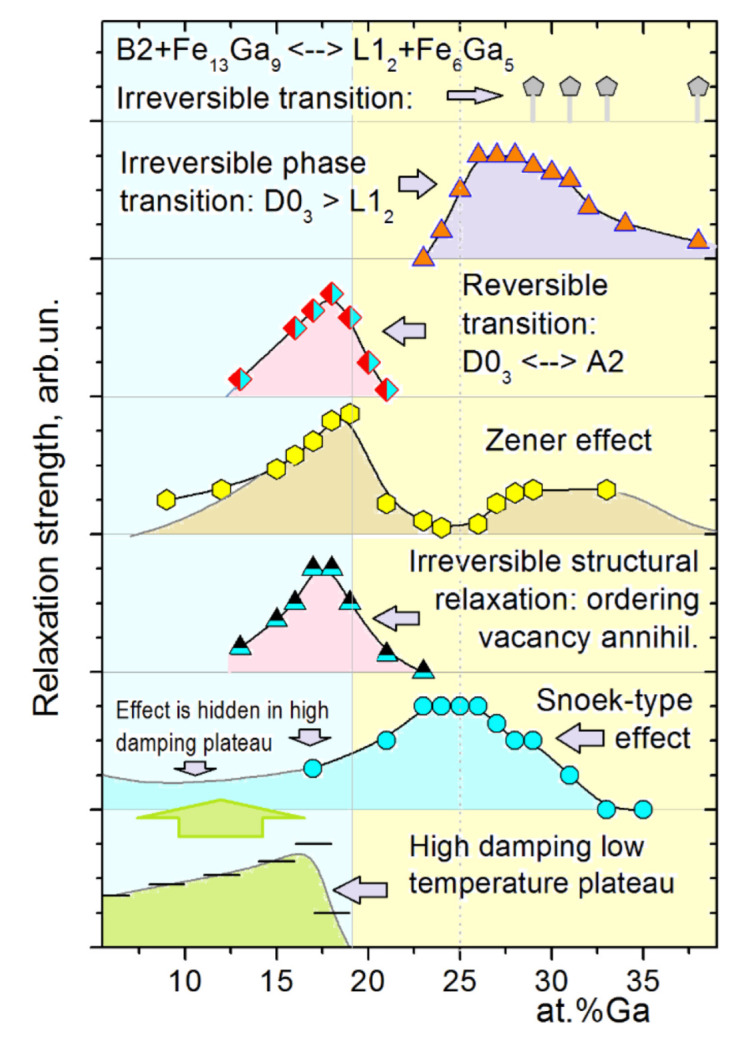
Scheme of different effects at temperature-dependent internal friction curves for rapidly cooled (water- or air-quenched and as-cast) Fe–(6–38)Ga: from bottom to the top—high-damping low-temperature plateau, Snoek-type thermally activated effect, first transient irreversible effect due to structural relaxation, Zener thermally activated peak, reversible transient effect due to the order–disorder transition, irreversible transient effect due to D0_3_ to L1_2_ phase transition, irreversible transient effect due to D0_3_ + Fe_13_Ga_9_ to L1_2_ + Fe_6_Ga_5_ phase transition. Values for relaxation strength for all effects are given in arbitrary units; they are supposed to provide qualitative but not quantitative dependencies. Light blue background underlines Fe–Ga alloys without first-order phase transition, while the light yellow background corresponds to the alloys with different first-order transitions. Vertical grey dotted line corresponds to the Fe_3_Ga composition.

In Figure 29, we present a sketch of most important and reliable temperature-dependent effects at heating in Fe–(6–38)Ga alloys measured for at least 4–5 different compositions. The main message from this sketch is to underline the concentration range of Ga content in Fe–Ga binary alloys for differently acting thermally activated and transient anelastic effects recorded at heating. At cooling after heating to 600 °C, all irreversible transient effects, Snoek-type relaxation, and high-damping low-temperature plateau do not exist at the experimental curves. In our scheme, we have not included the grain boundary relaxation and transient effects due to L1_2_ to D0_19_ and D0_19_ to B2 phases as they were measured only on a limited number of compositions (mainly with 27–28%Ga), as discussed above. The Snoek effect was definitely observed in the Fe–Ga alloys in the range of 0–32%Ga, but it strongly overlaps with high-damping plateau for the alloys with Ga < 19%, making proper evaluation of its activation parameters impossible; we show it in Figure 29 but only for the alloys with Ga > 19%.

Apart from binary Fe–Ga alloys, we discussed the effect of substituting Ga in Fe–Ga alloys by Al as well as the influence of rare earth elements on phase transitions and anelasticity in Fe–Ga based alloys. The substitution of Ga by Al atoms does not decrease the damping capacity of Fe–Ga alloys in contrast with the predictions from the Smith and Birchak model. Aluminium in Fe–Ga–Al alloys suppresses the formation of the L1_2_ phase (rare earth elements have the same effect), and, as a result, Al leads to the disappearance of the corresponding transient effect. Al may often lead to the formation of a two-headed Snoek peak as a result of carbon atom jumps on positions Fe-C-Ga and Fe-C-Al and influences of the parameters of the Zener relaxation by contributions from Al-Al, Ga-Ga, and Al-Ga atomic pairs.

Two issues about the microstructure of Fe–Ga alloys were not discussed in this review, supposing that they do not play an important role in the anelastic effects considered in this paper:

i. Several papers report intermediate steps for the D0_3_ to L1_2_ transition, which includes the appearance of the *m*-D0_3_, D0_22_, L6_0_, etc., nano or micro precipitations, and the twinning and local displacive character of this transition. In all cases, the appearance of these phases has a local character, and their volume fraction is very small to change the interpretation of anelastic effects discussed in this paper. Independent of the details of the transition mechanism, the related anelastic strain comes from the lattice deformation when the material is transformed. Phase transitions in the studied alloys are carefully evaluated by real-time neutron diffraction and discussed in this review;
ii. Moreover, above-mentioned local phases were reported only by either XRD or TEM methods, which study the surface of the samples. Several papers clearly demonstrated that the bulk and surface structure of Fe–Ga samples is rather different [13,148,149]. This is another reason why we exclude discussion on the details of the surface structure of Fe–Ga alloys as all anelastic effects were measured only on a bulk sample.

In this review paper, we report and discuss several thermally activated, transient, and hysteretic effects in Fe–Ga and Fe–Ga-based alloys along with analysis of alloy structure and phase transitions in bulk samples. Although the general ‘picture’ of anelastic effects is rather clear, several points remain open for further studies and discussions. Here, we would like to mention only three ‘hot’ examples:

1. The Zener effect in the alloys with the same composition but different structure (bcc or fcc);
2. Local mechanisms of Zener and Snoek effects in ternary Fe–Ga–Al alloys;
3. There are inconsistencies between significantly higher magnetostriction in Fe–Ga alloys compared with Fe–Al alloys and damping capacity of alloys of these systems.

We hope that analysis of anelasticity in the Fe–Ga-based alloys given in this paper will stimulate further studies.

## Figures and Tables

**Table 1:** Apparent values of the activation parameters for the P2 Zener relaxation in Fe–(15–21)Ga alloys [59].

Alloy	H, eV	τ_0_, s	β_τ_ (1 Hz)
Fe–15.5Ga	2.49 ± 0.05	4 × 10^−18^	1.6
Fe–16.5Ga	2.56 ± 0.02	9 × 10^−19^	1.7
Fe–17.5Ga	2.39 ± 0.05	8 × 10^−18^	1.9
Fe–18.6Ga	2.56 ± 0.02	6 × 10^−19^	2.1
Fe–19.5Ga	2.60 ± 0.06	9 × 10^−19^	2.2
Fe–20.7Ga	2.38 ± 0.02	5 × 10^−18^	2.0

**Table 2:** Mean values of the activation parameters for thermally activated P1 and P2 peaks in the Fe–19Ga–RE alloys [59].

Alloy	P1 (Snoek-Type)		P2 (Zener)	
	H, eV	τ_0_, s	(H), eV	τ_0_, s
Fe–18Ga–0.2La	HDP plateau instead of peak		2.40 ± 0.20	8 × 10^−18^
Fe–18.6Ga–0.1Dy	1.2 ± 0.2	7 × 10^−17^	2.60 ± 0.10	4 × 10^−19^
Fe–19.5Ga–0.15Tb	unclear		2.24 ± 0.06	6 × 10^−17^
Fe–18.5Ga–0.2Er	1.2 ± 0.1	8 × 10^−18^	2.70 ± 0.10	7 × 10^−20^
Fe–18.7Ga–0.1Yb	1.1 ± 0.1	4 × 10^−15^	2.50 ± 0.10	9 × 10^−19^

**Table 3:** Apparent values of activation energies (H) and distribution relaxation time (β_τ_) for Zener relaxation in Fe–(15–21)Ga alloys [49,59,60]. Values are rounded to tenths. In the alloys with 24.5 and 25.5%Ga, the peak height is very low and evaluation of the activation parameters for these two alloys is not precise.

	Ga, at.%	23.1	23.8	24.5	25.5	26.9	28.1	28.9	31.1
Snoek	H, eV	1.1	1.1	1.1	1.1	1.1	1.2	1.1	1.2
	τ_0_, s	10^−15^	10^−15^	10^−15^	10^−15^	10^−15^	10^−16^	10^−15^	10^−16^
Zener	H, eV	2.2	1.9	2.3	2.4	1.7	1.8	1.8	1.4
	τ_0_, s	10^−16^	10^−15^	-	-	10^−15^	10^−15^	10^−16^	10^−16^

**Table 4:** Values for ‘*n*’ in eqs ${\left(\frac{\dot{T}}{\omega }\right)}^{n}$ for P_Tr1_, P_Tr2_, and P_Tr2_ transitory peaks in Fe–27G at torsion vibrations.

	P_Tr1_ D0_3_ → L1_2_	P_Tr2_ L1_2_ → D0_19_	P_Tr3_ D0_19_ → A2(B2)
${\dot{T}}^{n}$	0.35	0.18	0.29
${1/\omega }^{n}$	0.41	0.22	0.44

**Table 5:** The coefficient (*n*) in power dependence Q_Tr_^−1^~Ṫ/f^n^ for the transient peaks in Fe–(16–38%)Ga as measured for forced bending vibrations.

Transition	Second-Order Transition Disorder (D0_3_ to A2)	First-Order Irreversible Transition (D0_3_ to L1_2_)	First-Order Irreversible Transition: From D0_3_ + Fe_13_Ga_9_ to L1_2_ + Fe_6_Ga_5_
Range of n	*n* ≈ 0.7–0.9	*n* ≈ 0.6 (average)	*n* ≈ 0.2–0.7
**% Ga**	**16–20%**	**24–28%**	**29–33% or 38%**
Fe–16.5Ga	0.91	-	-
Fe–17.5Ga	0.90	-	-
Fe–18.6Ga	0.69	-	-
Fe–19.5Ga	0.71	-	-
Fe–25.0Ga	-	0.57	-
Fe–25.5Ga	-	0.56	-
Fe–26.1Ga	-	0.62	-
Fe–26.9Ga	-	0.52	-
Fe–27.3Ga	-	0.63	-
Fe–27.6Ga	-	0.68	-
Fe–28.1Ga	-	0.61	-
Fe–28.9Ga	-	-	0.25
Fe–31.1Ga	-	-	0.69
Fe–32.9Ga	-	-	0.68
Fe–38.4Ga	-	-	0.21

**Table 6:** Temperature ranges of the existence of individual phases in Fe–Ga alloys during heating up to 850 °C. The temperature of the Fe_13_Ga_9_ volume fraction jump is given in parentheses.

Alloy	D0_3_/B2	Fe_13_Ga_9_	L1_2_	α-Fe_6_Ga_5_
Fe–31.1Ga	25–850	(415)–570,	470–650	570–670
Fe–32.9Ga	25–850	25 (390)–570	480–650	570–670
Fe–34.4Ga	25–850	25 (350)–580	500–670	560–700
Fe–38.4Ga	25–850	25–570	570–660	570–720
